# Overexpression of Peroxidase Gene *GsPRX9* Confers Salt Tolerance in Soybean

**DOI:** 10.3390/ijms20153745

**Published:** 2019-07-31

**Authors:** Ting Jin, Yangyang Sun, Ranran Zhao, Zhong Shan, Junyi Gai, Yan Li

**Affiliations:** National Key Laboratory of Crop Genetics and Germplasm Enhancement, National Center for Soybean Improvement, Key Laboratory for Biology and Genetic Improvement of Soybeans (General, Ministry of Agriculture), Jiangsu Collaborative Innovation Center for Modern Crop Production, Nanjing Agricultural University, Nanjing 210095, China

**Keywords:** *GsPRX9*, hairy roots, peroxidase, salt tolerance, soybean

## Abstract

Peroxidases play prominent roles in antioxidant responses and stress tolerance in plants; however, their functions in soybean tolerance to salt stress remain unclear. Here, we investigated the role of a peroxidase gene from the wild soybean (*Glycine soja*), *GsPRX9*, in soybean tolerance to salt stress. *GsPRX9* gene expression was induced by salt treatment in the roots of both salt-tolerant and -sensitive soybean varieties, and its relative expression level in the roots of salt-tolerant soybean varieties showed a significantly higher increase than in salt-sensitive varieties after NaCl treatment, suggesting its possible role in soybean response to salt stress. *GsPRX9*-overexpressing yeast (strains of INVSc1 and G19) grew better than the control under salt and H_2_O_2_ stress, and *GsPRX9*-overexpressing soybean composite plants showed higher shoot fresh weight and leaf relative water content than control plants after NaCl treatment. Moreover, the *GsPRX9*-overexpressing soybean hairy roots had higher root fresh weight, primary root length, activities of peroxidase and superoxide dismutase, and glutathione level, but lower H_2_O_2_ content than those in control roots under salt stress. These findings suggest that the overexpression of the *GsPRX9* gene enhanced the salt tolerance and antioxidant response in soybean. This study would provide new insights into the role of peroxidase in plant tolerance to salt stress.

## 1. Introduction

Soil salinization is an important abiotic factor affecting agricultural production and the environment [1]. Soil salinization can cause decreased soil osmotic potential, ion imbalance, disrupted physiological processes, and inhibited growth and development of plants, leading to reduced crop quality and yield, and even plant death in areas with severe salt–alkali stress [1,2]. Plants adapt to salt stress by three main mechanisms: osmotic stress tolerance, ion exclusion, and tissue tolerance [1]. Soybean plants experience two stages of salt stress: in the early stage, the young leaves of soybean seedlings wilt due to water loss in the initial few hours of salt stress; then soybean plants produce molecular substances that participate in osmotic regulation to help the wilted leaves recover [2]. In the second stage, Na^+^ accumulation affects photosynthesis and damages the leaves [1,2,3]. Soybean tolerance to salt stress is controlled by multiple genes. Overexpression of *GmCHX1* improved salt tolerance by regulating Na^+^/K^+^ balance in soybeans under salt stress [3]. A salt-responsive gene, *GmPIP1;6*, may be a multifunctional aquaporin involved in root water transport [4]. Overexpression of *GmbZIP1* enhanced plant tolerance to salt stress [5]. More genes related to salt tolerance in soybeans need to be discovered.

Peroxidases with different structures play various roles in diverse organisms [6,7]. Phylogenetically, the peroxidases fall into three classes (class I–III) of proteins [6,8]. Class I peroxidases, including cytochrome-c peroxidases (CcPs), catalase peroxidases (CPs), ascorbate peroxidases (APxs), and the hybrid APx–CcPs, are the most abundant peroxidases in plants, fungi, and prokaryotes [7,9]. These peroxidases are not glycosylated and do not have signal peptides, calcium ions, or disulfide bridges [9]. Class II peroxidases are fungal secretory peroxidases, primarily comprising lignin peroxidases (LiPs), manganese-dependent peroxidases (MnPs), versatile peroxidases (VPs), and other secreted fungal heme peroxidases [10,11]. Class III peroxidases (Prxs), are a group of secretory glycoproteins that have been found in all green plants [8,12].

Prxs are classical peroxidases and play key roles in many plant physiological functions, either by forming stable plant structures or by adapting the organisms to a more oxygenated environments [13,14]. Prxs have been confirmed to catalyze hydrogen peroxide (H_2_O_2_) oxidoreduction by transferring electrons from various donor molecules [12,15,16,17]. Due to their nonspecific reactions with different hydrogen donors, Prxs participate in diverse plant processes [15,16,18] such as auxin metabolism [19], lignin biosynthesis [12], suberin metabolism [20], cell wall elongation [21], seed coat mucilage extrusion [22], stress tolerance [23], testa/endosperm rupture [24], and pathogen defense [25]. Overexpression of *AtPRX3* enhanced plant salt tolerance [23]. *AtPRX17* affected the lignin and xylan accumulation in the cell wall via reactive oxygen species (ROS) signaling [12]. A recent study suggests that some Prxs were involved in housekeeping functions to scavenge ROS and H_2_O_2_ in rice [26].

In our previous transcriptomic study (unpublished) using a salt-tolerant wild soybean variety, we identified a salt-responsive gene (corresponding to Glyma.14g070800 in the reference genome of soybean variety Williams 82), which encodes a Class III peroxidase, and is designated as *GsPRX9*. Here we investigate the role of *GsPRX9* in salt tolerance by comparing the relative expression level of *GsPRX9* gene in salt-tolerant and -sensitive soybean varieties, and overexpression of *GsPRX9* in soybean hairy roots, composite plants and yeast (strains of INVSc1 and G19), respectively. The salt tolerance mechanism of *GsPRX9* was investigated by analyzing the activities of peroxidase (POD), superoxide dismutase (SOD), H_2_O_2_ content, glutathione (GSH) level, and the expression levels of potential oxidation-reduction related genes in *GsPRX9*-overexprssing soybean hairy roots under salt stress, to demonstrate its positive roles in scavenging ROS under salt stress. This study aims to provide new insights into the role of Prxs in soybean tolerance to salt stress and the possible underlying mechanisms.

## 2. Results

### 2.1. Molecular Characteristics of GsPRX9

**Figure 1:**
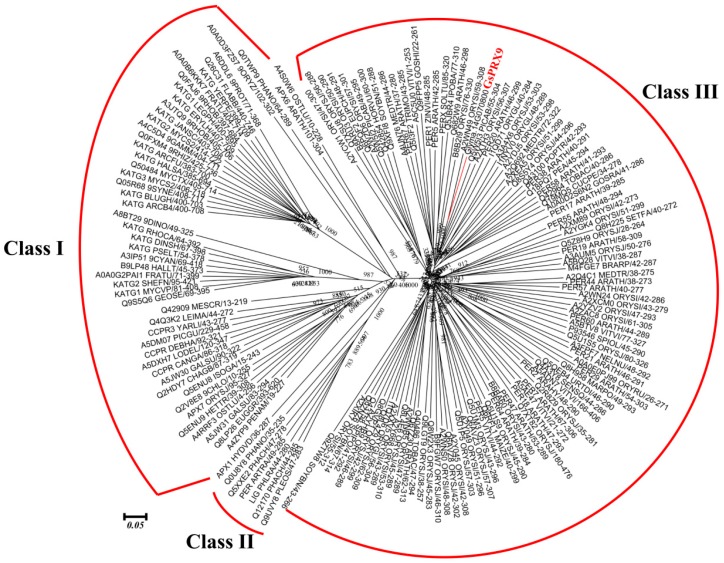
Phylogenetic tree of the 159 seed sequences of peroxidases superfamily and GsPRX9. The 159 seed sequences of peroxidases protein superfamily were downloaded from PFAM and the protein sequence of soybean PRX9 was downloaded from the Phytozome database. Unrooted phylogenetic tree was constructed using MEGA6.0 based on the Maximum Likelihood (ML) algorithm with 1000 bootstraps.

The coding sequence (CDS) of *GsPRX9* from the wild soybean (*G. soja*) in this study was found to be identical to the sequence of Glyma.14g070800 from the soybean reference genome (https://phytozome.jgi.doe.gov/pz/portal.html), which is 1035 bp in length and encodes 344 amino acids with a peroxidase domain consisting of 247 amino acids. Phylogenetic analysis of soybean PRX9, together with the 159 seed sequences (with 201 to 350 amino acids in length) of the peroxidases protein superfamily (Table S1), was performed. According to the functional annotation (Table S2) and classification [8] of the peroxidases superfamily, these 160 proteins were grouped into three classes (Figure 1). GsPRX9 was classified into the Class III peroxidases (Prxs).

### 2.2. Relative Expression of GsPRX9 in Response to NaCl in Salt-Tolerant and -Sensitive Soybean Varieties

**Figure 2:**
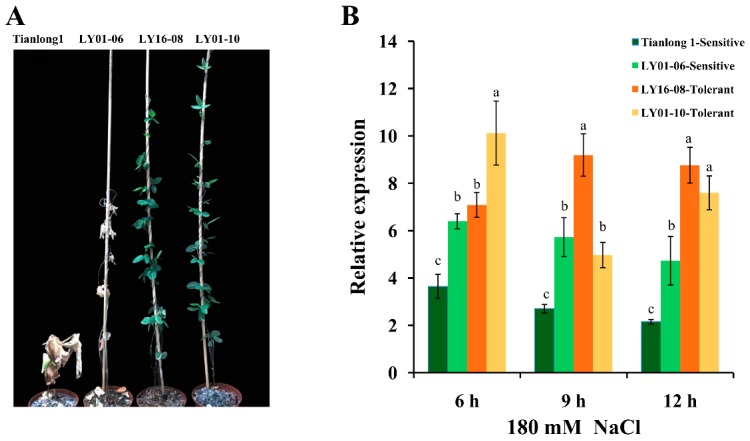
Phenotype and relative expression of *GsPRX9* in the roots of four soybean varieties under salt stress. (**A**) Phenotypes of four soybean varieties at seven days after 180 mM NaCl stress. (**B**) Relative expression of *GsPRX9* in response to salt stress. Soybean seedlings were treated with 0 or 180 mM NaCl for 6 h, 9 h, and 12 h and the root samples were collected. Roots receiving 0 mM NaCl treatment at each time point were used as controls. Data represent mean and standard deviation of three repeats (*n* = 3). Data with the same letters in lowercases (a, b, and c) above bars indicate no significant differences at the 0.05 level between soybean varieties at each time point according to Duncan’s multiple range test.

After seven days of 180 mM NaCl stress, the phenotypic difference between the four soybean varieties was obvious (Figure 2A): the leaves of Tianlong1 and “LY01-06” became yellow and dead, and therefore are salt-sensitive soybean varieties; the leaves of “LY16-08” and “LY01-10” were still green and normal, and so belong to salt-tolerant soybean varieties. The relative expression levels of *GsPRX9* in response to salt stress (180 mM NaCl) in these four soybean varieties were investigated using qRT-PCR. *GsPRX9* gene expression was induced by 180 mM NaCl treatment in the roots of all soybean varieties (Figure 2B). The relative expression level of *GsPRX9* gene in the roots of “LY16-08” (salt-tolerant) showed a significantly (*p* < 0.05 by Duncan’s multiple range test) greater increase at 9 and 12 h after salt treatment than the two salt-sensitive soybean varieties, and there was significantly (*p* < 0.05 by Duncan’s multiple range test) greater upregulation in the roots of “LY01-10” (salt-tolerant) at 6 and 12 h in response to salt stress than the two salt-sensitive soybean varieties. These results suggested that *GsPRX9* might play important roles in the soybean response to salt stress.

### 2.3. Overexpression of GsPRX9 Improved Yeast Tolerance to NaCl and H_2_O_2_ Stress

**Figure 3:**
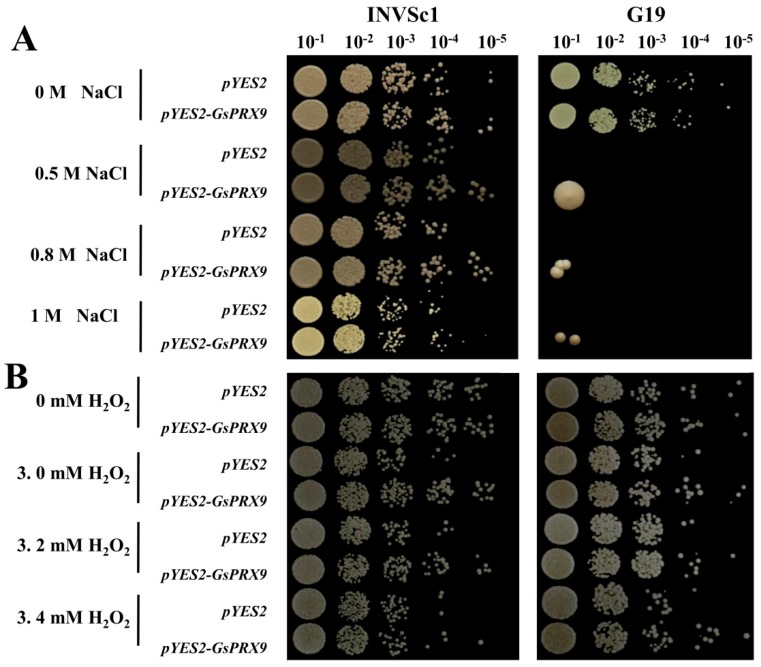
Effect of *GsPRX9* overexpression on the tolerance of yeast strains of INVSc1 and G19 to salt (**A**) and H_2_O_2_ (**B**) stress. pYES2 represents the yeast with empty vector and pYES2–*GsPRX9* represents the yeast overexpressing *GsPRX9*. Photos were taken after 72 h of incubation at 30 °C. The dilution rates of YPD medium containing the yeast transformants were 10^−1^, 10^−2^, 10^−3^, 10^−4^, and 10^−5^.

The growth characteristics of yeast (strains of INVSc1 and G19), containing pYES2-*GsPRX9* plasmid or pYES2 empty vector, respectively, on a yeast extract-peptone–dextrose (YPD) medium with different concentrations of NaCl (0, 0.5, 0.8, and 1 M) or H_2_O_2_ (0, 3, 3.2, and 3.4 mM) were compared. There was no obvious difference between the yeast containing pYES2-*GsPRX9* and the yeast with pYES2 empty vector on normal (0 M NaCl and 0 mM H_2_O_2_) YPD medium (Figure 3). The pYES2-*GsPRX9*-transformed INVSc1 yeast grew better than the pYES2 empty-vector-transformed INVSc1 yeast on a YPD medium containing 0.5 or 0.8 M NaCl (Figure 3A). In addition, when the salt-sensitive yeast mutant G19 was used, pYES2-*GsPRX9*-transformed yeast showed better growth than the pYES2 empty-vector-transformed G19 yeast on YPD containing 0.5, 0.8, or 1 M NaCl (Figure 3A). Under H_2_O_2_ stress, pYES2-*GsPRX9*-transformed yeast (strains of INVSc1 and G19) exhibited better growth than the pYES2 empty-vector-transformed yeast (Figure 3B). These results revealed that overexpression of *GsPRX9* improved the tolerance of INVSc1 and G19 yeasts to NaCl and H_2_O_2_ stress.

### 2.4. GsPRX9-Overexpressing Soybean Composite Plants Showed Better Tolerance to NaCl Stress

**Figure 4:**
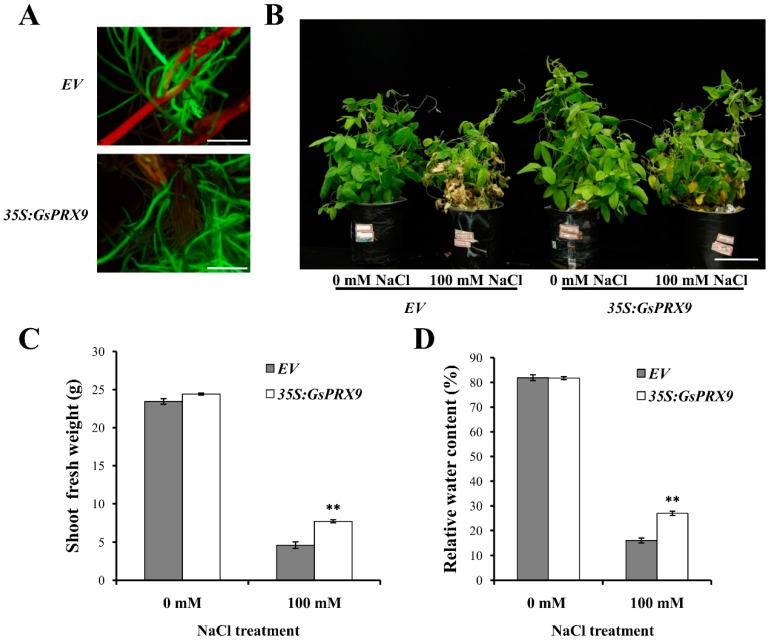
Salt tolerance analyses of transgenic soybean composite plants. (**A**) Identification of positive transgenic soybeans by green fluorescence using a stereoscopic fluorescence microscope. Bar = 1 cm. (**B**–**D**) Phenotype, shoot fresh weight, and leaf relative water content of transgenic soybean composite plants under 0 mM or 100 mM NaCl for seven days, respectively. Bar = 5 cm. *EV* represents plants with the empty vector pBinGFP4 and *35S:GsPRX9* represents plants with the recombinant vector pBinGFP4–*GsPRX9* (*GsPRX9* was expressed in fusion with GFP, driven by a CaMV 35S promoter). Data represent the mean ± standard deviation of three biological replications and each repeat contained 10 independent transgenic plants (*n* = 3 × 10 = 30). ** represents significant difference between *EV* and *35S:GsPRX9* under the same conditions at the 0.01 level by Student’s *t*-test.

The transgenic soybean composite plants were obtained by *Agrobacterium rhizogenes* K599-mediated transformation of pBinGFP4–*GsPRX9* or pBinGFP4 (empty vector, containing the green fluorescence protein, GFP) into soybean hypocotyls, to investigate the effect of *GsPRX9* overexpression on the salt tolerance of soybean plants. The positive transgenic soybean composite plants (indicated by green fluorescence, Figure 4A) overexpressing *GsPRX9* or the empty vector pBinGFP4 with similar root length and root volume were treated with 0 or 100 mM NaCl for seven days. The composite plants showed better growth under 0 mM NaCl than those under 100 mM NaCl treatment (Figure 4B). Under 100 mM NaCl treatment, the soybean composite plants with *GsPRX9* overexpression showed better growth than composite plants with the empty vector pBinGFP4 (Figure 4B and Figure S1A–C). The average shoot fresh weight and leaf relative water content of *GsPRX9*-overexpressing soybean composite plants were significantly (*p* < 0.01 by *t*-test) higher than those of composite plants with the empty vector under 100 mM NaCl treatment (Figure 4C,D and Figure S1D–I). No significant differences (*p* > 0.05 by *t*-test) were found in shoot fresh weight and leaf relative water content between *GsPRX9*-overexpressing plants and plants with empty vector under normal condition (0 mM NaCl). These results demonstrated that overexpression of *GsPRX9* could improve the tolerance of soybean plants to salt stress.

### 2.5. Overexpression of GsPRX9 Enhanced the Salt Tolerance of Soybean Hairy Roots

**Figure 5:**
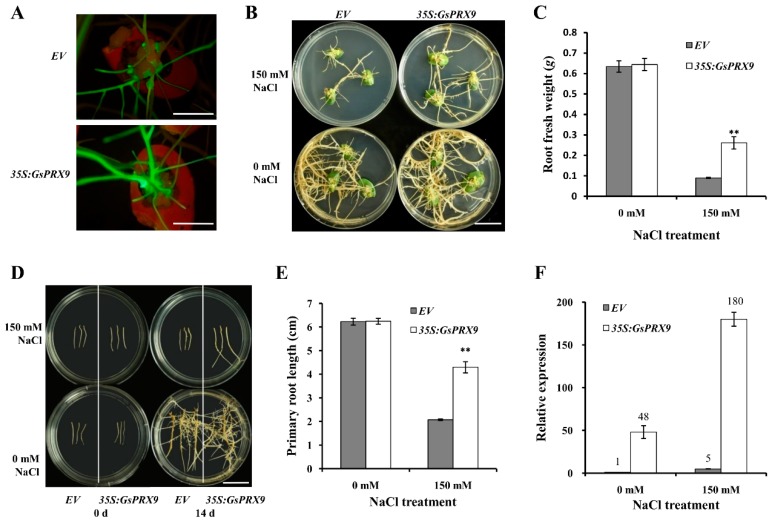
Salt tolerance analyses of transgenic soybean hairy roots. (**A**) Identification of positive transgenic soybean hairy roots by green fluorescence using a stereoscopic fluorescence microscope. Bar = 1 cm. (**B**,**C**) Phenotype and root fresh weight of transgenic soybean cotyledon hairy roots receiving 0 or 150 mM NaCl treatment for 14 days, respectively. Bar = 1 cm. (**D**,**E**) Phenotype and primary root length of re-generated transgenic soybean hairy roots under 0 or 150 mM NaCl treatment for 14 days, respectively. Bar = 1 cm. (**F**) The relative expression level of *GsPRX9* in transgenic soybean hairy roots at 24 h after 0 or 150 mM NaCl treatment. *EV* represents roots with the empty vector pBinGFP4, and *35S:GsPRX9* represents roots with the recombinant vector pBinGFP4-*GsPRX9* (*GsPRX9* was expressed in fusion with GFP, driven by a CaMV 35S promoter). The relative expression level of *GsPRX9* in transgenic soybean hairy roots was in comparison to that in soybean hairy roots containing the empty vector under 0 mM NaCl (control). Data represent the mean ± standard deviation of four biological replications and each repeat contained at least three independent transgenic hairy roots (*n* ≥ 12). ** represents significant difference between *EV* and *35S:GsPRX9* under the same condition at the 0.01 level by Student’s *t*-test.

To further confirm the effect of *GsPRX9* overexpression on salt tolerance of soybean, we transformed soybean cotyledons with *A. rhizogenes* K599 containing pBinGFP4-*GsPRX9* plasmid or the empty vector pBinGFP4. After 14 days of 150 mM NaCl treatment, the soybean hairy roots overexpressing *GsPRX9* showed better growth than the control (hairy roots transformed by the empty vector pBinGFP4; Figure 5A,B and Figure S2A–D). In comparison with the hairy roots transformed by the empty vector, the fresh weight of *GsPRX9*-overexpressing soybean hairy roots was significantly (*p* < 0.01 by *t*-test) higher under 150 mM NaCl treatment (Figure 5C and Figure S2E–H), while no significant difference (*p* > 0.05 by *t*-test) was observed under 0 mM NaCl conditions.

Furthermore, we investigated the effect of *GsPRX9* overexpression on the re-generated soybean hairy roots in response to salt stress. Root tips of 3 cm in length were cut off from the positive hairy roots with pBinGFP4-*GsPRX9* or pBinGFP4, respectively, and transferred on the medium containing 0 mM or 150 mM NaCl to grow for 14 days (Figure 5D and Figure S3A–D). The average primary root length of soybean hairy roots overexpressing *GsPRX9* was significantly (*p* < 0.01 by *t*-test) longer than that of the hairy roots containing the pBinGFP4 empty vector grown on a medium containing 150 mM NaCl (Figure 5E and Figure S3E–H), but no obvious difference was observed for soybean hairy roots grown on a medium containing 0 mM NaCl. By qRT-PCR, we confirmed that the relative expression level of *GsPRX9* in soybean hairy roots transformed by pBinGFP4-*GsPRX9* was much higher than the level in soybean hairy roots transformed by a pBinGFP4 empty vector, under either 0 or 150 mM NaCl treatment (Figure 5F and Figure S3I–L). These results suggest that overexpression of *GsPRX9* enhanced the salt tolerance of soybean hairy roots.

### 2.6. Overexpression of GsPRX9 Enhanced the Antioxidant Response in Soybean Hairy Roots

**Figure 6:**
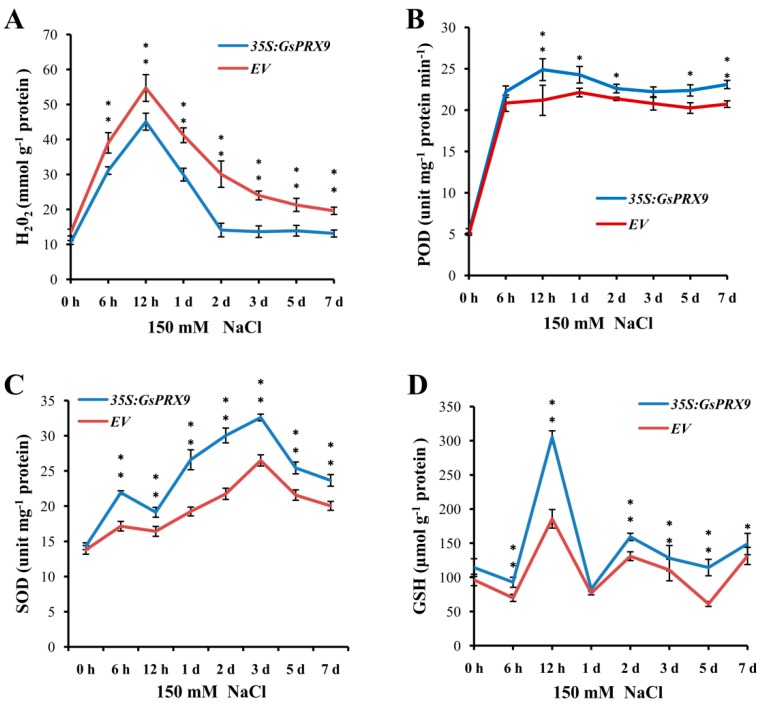
Effect of *GsPRX9* overexpression on H_2_O_2_ content (**A**), POD activity (**B**), SOD activity (**C**), and GSH level (**D**) in transgenic soybean hairy roots under salt stress. *EV* represents soybean hairy roots with the empty vector pBinGFP4, and *35S:GsPRX9* represents roots with the recombinant vector pBinGFP4–*GsPRX9* (*GsPRX9* was expressed in fusion with GFP, driven by a CaMV 35S promoter). The content of hydrogen peroxide (H_2_O_2_), activity of peroxidase (POD), superoxide dismutase (SOD), and glutathione level (GSH) in soybean hairy roots were measured at 0 h, 6 h, 12 h, 1 d, 2 d, 3 d, 5 d, and 7 d after 150 mM NaCl treatment. Data represent the mean ± standard deviation of four biological replications and each repeat contained three independent transgenic hairy roots (*n* = 4 × 3 = 12). * and ** represents a significant difference between *EV* and *35S:GsPRX9* for each time point at the 0.05 or 0.01 level by Student’s *t*-test, respectively.

The annotation of *GsPRX9* suggests it is involved in the oxidation–reduction process and the response to oxidative stress, and overexpression of *GsPRX9* gene enhanced the salt tolerance of soybean hairy roots; therefore, we proposed that overexpression of *GsPRX9* might enhance the antioxidant responses of soybean hairy roots under salt stress. To investigate this, we measured the content of H_2_O_2_ (Figure 6A and Figure S4A–D), the activity of POD (Figure 6B and Figure S4E–H), the activity of SOD (Figure 6C and Figure S4I–L), and the GSH level (Figure 6D and Figure S4M–P) in transgenic soybean hairy roots at 0 h, 6 h, 12 h, 1 d (day), 2 d, 3 d, 5 d, and 7 d after 150 mM NaCl treatment. The H_2_O_2_ content increased rapidly with the duration of salt stress at early time points, and reached a peak at 12 h, then decreased during 12 h to 2 d of salt stress, and remained relative low levels after 2 d (Figure 6A). Correspondingly, the POD activity also increased rapidly at early time points (6 h) and reached a peak at 12 h or 1 d, following with relatively stable levels after 1 d until the end of the experiment (Figure 6B). The SOD activity showed a relatively steady increase after salt stress and reached a peak at 3 d, then decreased after 3 d (Figure 6C). The GSH level peaked at 12 h after salt treatment (Figure 6D). As expected, we found that *GsPRX9*-overexpressing soybean hairy roots had lower H_2_O_2_ content (Figure 6A and Figure S4A–D), but higher POD and SOD activity, as well as higher GSH level than those in the soybean hairy roots with the empty vector pBinGFP4 (Figure 6B–D and Figure S4E–P). The above results suggest that *GsPRX9* participates in the ROS scavenging process, and overexpression of *GsPRX9* can improve the activities of POD and SOD, and the GSH level, thus enhancing the antioxidant response and salt tolerance of soybean hairy roots by manipulating the ROS balance during salt stress.

## 3. Discussion

### 3.1. GsPRX9 Overexpression Can Improve the Tolerance of Yeasts and Soybeans to NaCl Stress

Plant growth and development may be influenced by biotic and abiotic stresses, such as insects, drought, cold, and high salinity [27]. In this study, a peroxidase gene from the wild soybean, *GsPRX9*, was found to be involved in the salt tolerance of soybean. *GsPRX9* expression can be induced by NaCl treatment (Figure 2), suggesting its possible role in soybean tolerance to salt stress. In order to quickly test if *GsPRX9* plays a role in salt tolerance, we overexpressed *GsPRX9* in yeasts. Yeasts have been used to study the functions of plant genes in salt tolerance [28,29]. Overexpression of *GmCLC1* can enhance the survival rate of yeast *gef1* mutant with different chloride salts (MnCl_2_, KCl, NaCl) [28]. Yeast overexpressing *OsLOL5* exhibited better growth than yeast transformed with the empty vector pYES2 [29]. In this work, *GsPRX9*-overexpressing yeasts grew better than yeasts transformed by the empty vectors under NaCl and H_2_O_2_ (Figure 3). Therefore, *GsPRX9* overexpression can enhance yeast (strains of INVSc1 and G19) tolerance to salt and H_2_O_2_ stress.

Next, we confirmed the effect of *GsPRX9* on salt tolerance in soybean hairy roots. Plant roots have evolved various defense mechanisms against salt stress at morphological, physiological and molecular levels, such as root architecture adaptation, osmotic adjustment, enhancement of antioxidant defense, maintenance of cell membrane stability, and expression of salt responsive genes and proteins [30,31,32,33,34,35]. Therefore, soybean hairy roots can be used to further verify the gene functions in salt tolerance. In a previous study, *PgTIP1* overexpressing soybean hairy roots displayed superior salt tolerance compared to the empty-vector-transformed ones [36]. In this study, we employed soybean hairy roots to investigate the role of *GsPRX9* in salt tolerance (Figure 5 and Figure S2). We found that overexpression of *GsPRX9* in soybean hairy roots increased the root fresh weight (Figure 5C and Figure S2E–H) and primary root length (Figure 5D,E and Figure S3A–H). *GsPRX9* overexpression can also protect the shoots from salt damage as demonstrated by the transgenic soybean composite plants (Figure 4 and Figure S1). Taken together, these results revealed that overexpression of *GsPRX9* gene enhanced the salt tolerance of soybean hairy roots and composite plants.

### 3.2. GsPRX9 Mediates the ROS Regulation Network

ROS plays a critical role in different plant species [37,38]. Prxs have the interesting capacity to both scavenge and produce ROS [39]. Increased ROS/H_2_O_2_ detoxification and osmotic adjustment in plants improved their tolerance to abiotic stresses [34,40,41,42,43]. It has been reported that class III peroxidases function as ROS regulators, co-localized with ROS production at the germination stage of *Arabidopsis thaliana*, and notably AtPRX07, are involved in controlling the H_2_O_2_ concentration [39]. *OsPrx24* functions as a ROS scavenger and is highly expressed in guard cells [26].

In our present study, lower H_2_O_2_ content (Figure 6A and Figure S4A–D), higher activities of POD (Figure 6B and Figure S4E–H) and SOD (Figure 6C and Figure S4I–L), and a higher GSH level were observed in *GsPRX9* overexpressing soybean hairy roots compared with soybean hairy roots transformed by the empty vector. POD and SOD are protective enzymes in organisms and play important roles in scavenging free radicals. SOD can convert O2^−^ to H_2_O_2_ [44,45], and POD removes H_2_O_2_ to generate H_2_O [46,47,48,49]. As a non-enzymatic antioxidant, GSH can also eliminate free radicals to protect plants from ROS damage [50]. The synergistic action of these molecules can maintain free radicals at a low level to avoid membrane damage and protect cells [46]. Thus, our results indicated that overexpression of *GsPRX9* could increase the activities of SOD and POD, and the GSH level, to enhance ROS scavenging capability when exposed to salt stress, which ultimately improves salt tolerance. In addition, other known antioxidant enzymes such as ascorbate peroxidase (APX), catalase (CAT), glutathione peroxidase (GPX), mono-dehydroascorbate reductase (MDHAR), dehydroascorbate reductase (DHAR), glutathione reductase (GR), and glutathione S-transferase (GST), could work together to detoxify ROS [51].

**Figure 7:**
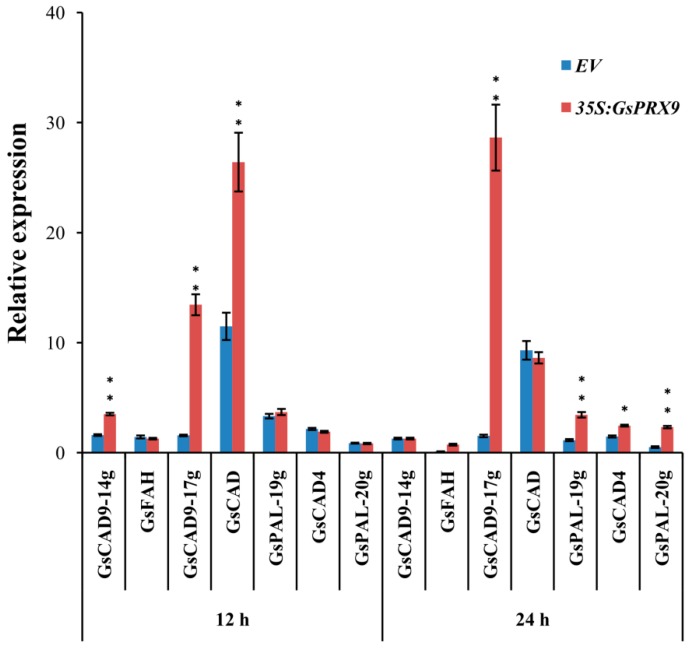
Relative expression of genes encoding potential GsPRX9-interacting proteins in soybean hairy roots. Root samples receiving 0 mM NaCl treatment at each time point were used as controls. Data represent mean ± standard deviation of four biological replications with three repeats within each replication (*n* = 4 × 3 = 12). *EV* represents soybean hairy roots with the empty vector pBinGFP4, and *35S:GsPRX9* represents roots with the recombinant vector pBinGFP4–*GsPRX9* (*GsPRX9* was expressed in fusion with GFP, driven by a CaMV 35S promoter). * and ** represents significant difference between *EV* and *35S:GsPRX9* for each time point at the 0.05 or 0.01 level by Student’s *t*-test, respectively.

Although the regulation network of oxidative balance is complicated, H_2_O_2_ is known as the key signaling molecule [52]. *GsPRX9* overexpressing soybean hairy roots can ultimately reduce the H_2_O_2_ content to protect soybean roots from ROS damage, probably through a complex antioxidant machinery in addition to the increased activities of SOD and POD, and the GSH level. It is interesting that, based on protein-protein interaction network analyses, GsPRX9 might interact with 10 other proteins (Figure S5), with five of them annotated as involved in the oxidation-reduction process (GO: 0055114) and two annotated as involved in the response to oxidative stress (GO: 0006979) (Table S3). The genes encoding these interacting proteins might be co-expressed with *GsPRX9* at the transcriptional level. Therefore, we performed qRT-PCR to determine the relative expression of these seven genes that might be related to oxidation–reduction or response to oxidative stress, including four *CAD* (encoding cinnamyl-alcohol dehydrogenase) genes, two *PAL* (encoding phenylalanine ammonia-lyase) genes, and one *FAH* (encoding Ferulate-5-hydroxylase) gene. The relative expression level of *GsCAD9-14g*, *GsCAD9-17g*, and *GsCAD* at 12 h and *GsCAD9-17g*, *GsPAL-19g*, *GsCAD4*, and *GsPAL-20g* at 24 h after salt treatment in the roots of *GsPRX9*-overexpressing hairy roots showed a significantly greater increase than in hairy roots containing the empty vector (Figure 7). These results suggest that the overexpression of *GsPRX9* increased the expression of other genes of soybean hairy roots in response to salt stress. Taken together, overexpression of *GsPRX9* enhanced soybean salt tolerance through mediation of the ROS regulatory network.

## 4. Materials and Methods

### 4.1. Plant Materials and Growth Conditions

Soybean seeds, including salt-tolerant (“LY01-10”, “LY16-08”) and -sensitive varieties (“LY01-06”, “Tianlong1”), were obtained from the National Center for Soybean Improvement, Nanjing Agricultural University, Nanjing, China. Soybean seeds were germinated in sterile nutrient soil (turf + vermiculite + perlite, 2:2:1) and grown in a greenhouse for 12 days, at 28 °C (day)/24 °C (night) with 14 h (light)/10 h (dark) photoperiod, and the soybean seedlings were then subjected to 0 or 180 mM NaCl treatment.

### 4.2. RNA Isolation and Gene Cloning

Total RNA was isolated from soybean roots using an RNAprep Pure Plant Kit (Tiangen Biotech, Beijing, China), and the first-strand cDNA was synthesized using a PrimeScript™ 1st Strand cDNA Synthesis Kit (TaKaRa, Dalian, China). Primers were designed by Primer Premier 5 software (Premier Biosoft International, Palo Alto, CA, USA), and the specificities of primers were analyzed by Primer-BLAST (Figure S6) (https://www.ncbi.nlm.nih.gov/tools/primer-blast/). The primers were synthesized at GenScript (Nanjing, China) and their sequences are listed in Table S4. The sequence of Glyma.14g070800 was downloaded from Phytozome (https://phytozome.jgi.doe.gov/pz/portal.html) and used as a reference to design primers for cloning *GsPRX9*. The full-length CDS of *GsPRX9* was amplified from the cDNA of a wild soybean variety (“LY01-10”) following the PCR protocol: 94 °C for 5 min; 35 cycles of 94 °C for 30 s, 58 °C for 30 s, and 72 °C for 2 min; and 72 °C for 5 min, in a 50 μL reaction mixture (600 μg of DNA, 0.25 mM of each primer (Table S4), 25 μL of 2 × Gflex PCR buffer and 1 unit of Tks Gflex DNA polymerase (TaKaRa, Dalian, China)), and sequenced by GenScript (Nanjing, China).

### 4.3. Phylogenetic Analysis of Soybean PRX9 and Other Peroxidase Proteins

The 159 seed sequences of the peroxidases protein superfamily (Table S1) were identified using the structural domain of “peroxidase” (PF00141) and downloaded from PFAM (http://pfam.xfam.org/family/PF00141.22#tabview=tab3). The full protein sequences of the above peroxidases and soybean PRX9 were used for multiple sequence alignments by ClustalW2 (https://www.ebi.ac.uk/Tools/msa/clustalw2/) [53]. The unrooted phylogenetic tree was then constructed using MEGA 6.0 [54], based on the Maximum Likelihood (ML) algorithm with 1000 bootstraps.

### 4.4. Quantitative Real-Time PCR (qRT-PCR) Analysis

Total RNA, cDNA, and primers were obtained as described above. The amplicon specificity was verified by melting curve analysis (Figure S7) and agarose gel electrophoresis. The qRT-PCR was performed on a Roche 480 Realtime detection system (Roche Diagnostics, Rotkreuz, Switzerland) following the manufacturer’s instructions, using SYBR Premix ExTaq II Mix (TaKaRa, Dalian, China) in a final volume of 15 μL containing 2 μL cDNA, 7.5 μL SYBR Premix ExTaq II (TaKaRa, Dalian, China), and 200 nM of forward and reverse primers. The amplification program was set as follows: initial denaturation at 95 °C for 5 min; 40 cycles of denaturation at 95 °C for 10 s, annealing at 58 °C for 20 s, and extension at 72 °C for 20 s. The experiments were performed in triplicate. The amplification efficiencies (E) of primer pairs were estimated by qRT-PCR using 1×, 5×, 10×, 20×, and 30× dilutions of cDNA, according to the equation: E = 10^−1/slope^−1 [55]. Primers and amplification efficiencies of qRT-PCR were shown in Table S4. *GmUKN1* was used as the reference gene [56], and the relatively constant Ct values of *GmUKN1* across all samples demonstrated its invariant expression under our experimental conditions (Table S5), suggesting that it is suitable for the internal control of qRT-PCR. The relative expression levels of test genes were analyzed by the 2^−^^△△C*t*^ methods [57].

### 4.5. Plasmid Construction and Genetic Transformation

The PCR products of *GsPRX9* with A-Tailing were cloned into a pMD19-T vector (TaKaRa, Dalian, China) using a One-Step Cloning Kit (Vazyme, Nanjing, China), and confirmed by sequencing. The CDS of *GsPRX9* was then cloned into pBinGFP4 [58] and pYES2 vectors (Vazyme, Nanjing, China), respectively, using the *Kpn* I and *Bam* HI (TaKaRa, Dalian, China) sites, to obtain the recombinant plasmids of pBinGFP4–*GsPRX9* (*GsPRX9* was expressed in fusion with GFP, driven by a CaMV 35S promoter) and pYES2–*GsPRX9*. After confirmation by sequencing, the recombinant pBinGFP4–*GsPRX9* plasmid and empty vector (pBinGFP4) were transformed into soybeans using *Agrobacterium* (*A. rhizogenes* K599)-mediated genetic transformation [59]. The pYES2–*GsPRX9* plasmid and empty vector (pYES2) were transformed into the *Saccharomyces cerevisiae* strain INVSc1 and salt-sensitive mutant strain G19 using the PEG/LiAc procedure (TaKaRa, Dalian, China) according to the manufacturer’s instructions.

### 4.6. Evaluation of the Tolerance of Yeast Strains of INVSc1 and G19 to Salt and H_2_O_2_ Stress

Yeasts were grown in a synthetic medium minus the appropriate amino acids (SD-Ura) to select the transformants, as previously described [29]. The positive transformants were screened by PCR [60], and then grown on s YPD medium (TaKaRa, Dalian, China) supplemented with different concentrations of NaCl (0, 0.5, 0.8, and 1 M) or H_2_O_2_ (0, 3, 3.2, and 3.4 mM) at 30 °C until an obvious phenotypic difference was observed between *GsPRX9* overexpressing yeast and the corresponding control yeast with empty vector [29].

### 4.7. Salt Tolerance Analysis Using Soybean Composite Plants

The seeds of salt-sensitive soybean “LY01-06” were germinated in sterile vermiculite with 1/2 Hoagland solution (pH = 6.5) for five days, then soybean hypocotyls were incised and infected by *A. rhizogenes* K599 containing pBinGFP4–*GsPRX9* or pBinGFP4, and kept in the dark for 24 h with the wound wrapped in aluminum foil to maintain high humidity. The soybean seedlings were then transferred to 1/2 Hoagland solution and grown under high humidity at 28 °C (day) / 24 °C (night) under a 14 h (light) / 10 h (dark) photoperiod for 14 days. Next, the positive transgenic soybean composite plants with pBinGFP4–*GsPRX9* or pBinGFP4 were identified by green fluorescence signals using a stereoscopic fluorescence microscope (Olympus DP72, Tokyo, Japan), and the negative roots were removed. Five days later, these plants were treated with 1/2 Hoagland solution containing 0 or 100 mM NaCl (pH = 6.5) for seven days. The shoot fresh weight and leaf relative water content [61] were measured at day seven post-treatment. Three biological replications were performed and 10 independent transgenic plants were measured for each repeat.

### 4.8. Salt Tolerance Analysis Using Soybean Hairy Roots

The salt tolerance evaluation of soybean hairy roots was performed according to previously published methods [3,62]. Seeds of salt-sensitive soybean variety “Tianlong1” were surface-sterilized and placed onto MS medium (pH = 6.5) for germination. The fully developed cotyledons were cut in half and incised with sterile scalpels, and then co-cultivated with *A. rhizogenes* K599 (containing pBinGFP4–*GsPRX9* plasmid or pBinGFP4) on White solid medium (MDBio, pH = 5.8) for 15 days without light. Positive transgenic soybean hairy roots were identified by green fluorescence signals with a stereoscopic fluorescence microscope (Olympus DP72, Tokyo, Japan). The soybean cotyledons with all hairy roots shown as positives were then transferred to the White solid medium (MDBio, pH = 5.8) with 0 mM (control) and 150 mM NaCl containing 500 mg/L carbenicillin and 50 mg/L cefotaxime (Sangon Biotech, Shanghai, China) at 22 °C for 14 days. Root fresh weight was recorded at day 14 post-treatment. To regenerate positive transgenic roots, the soybean hairy roots with green fluorescence signals were selected and their root tips (3 cm in length) were cut, and then transferred onto White solid medium (pH = 5.8, including carbenicillin 500 mg/L and cefotaxime 50 mg/L) containing 0 or 150 mM NaCl. Roots were sampled at 24 h post-treatment for detection of the *GsPRX9* gene expression. The positive hairy roots continued to grow until day 14, and the lengths of primary hairy roots were measured. Four biological replications were performed and at least three hairy roots were measured for each repeat.

### 4.9. Measurement of POD and SOD Activities, H_2_O_2_ Content, and GSH Level

The activities of POD and SOD, H_2_O_2_ content, and GSH level in soybean hairy roots were measured as described previously [63,64]. In brief, total proteins were extracted using the vegetable protein extraction kit (KeyGene BioTECH, Nanjing, China) and quantified using a BCA protein assay kit (KeyGene BioTECH). The activities of POD and SOD, H_2_O_2_ content, and GSH level were measured using a peroxidase kit (Jiancheng BioTECH, Nanjing, China), superoxide dismutase kit (Keming BioTECH, Suzhou, China), H_2_O_2_ test kit (Jiancheng BioTECH, Nanjing, China), and GSH test kit (Jiancheng BioTECH, Nanjing, China) according to the instructions from the manufacturers and previously published methods [63,64]. All measurements were performed with four biological replications and three independent soybean hairy roots were used in each replication.

### 4.10. Protein–Protein Interaction Network Analysis

In order to identify the potential GsPRX9 interacting proteins that might be helpful to understand the function of *GsPRX9*, the Search Tool for the Retrieval of Interacting Genes (STRING, https://string-db.org/) was employed to predicate the protein–protein interaction (PPI) network, using the combined_score ≥ 0.9. Cytoscape software (http://www.cytoscape.org/) [65] was used for the visualization of protein–protein interaction network. Annotations of the potential GsPRX9 interacting proteins were obtained from the gene ontology (GO) database (http://www.geneontology.org/).

### 4.11. Statistical Analysis

All experiments were performed at least in triplicate, and the results were reported as mean ± standard deviation (SD). Statistical analyses were performed using SAS 9.2 (SAS Institute Inc., Cary, NC, USA). Differences between the two groups were analyzed using Student’s *t*-tests, while differences among more than two groups were analyzed using Duncan’s multiple range tests.

## 5. Conclusions

To conclude, *GsPRX9* encodes a peroxidase protein that was induced by salt stress, and its upregulation was higher in salt-tolerant than in salt-sensitive soybean varieties. Overexpression of *GsPRX9* enhanced the salt tolerance of yeast (strains of INVSc1 and G19), soybean hairy roots, and soybean composite plants. The root fresh weight, primary root length, activities of POD and SOD, as well as GSH level in soybean hairy roots overexpressing *GsPRX9* were greater than those in controls (empty vector). Taken together, these findings suggest that *GsPRX9* plays an important role in soybean tolerance to salt stress.

## Supplementary Materials

The following are available online at https://www.mdpi.com/1422-0067/20/15/3745/s1.

## Abbreviations

APxs	Ascorbate peroxidases
CcPs	Cytochrome-c peroxidases
CDS	Coding sequence
CPs	Catalase peroxidases
GSH	Glutathione
GsPRX9	Peroxidase 9 from the wild soybean (*Glycine soja*)
H_2_O_2_	Hydrogen peroxide
LiPs	Lignin peroxidases
MnPs	Manganese-dependent peroxidases
PCR	Polymerase chain reaction
POD	Peroxidase
Prxs	Class III peroxidases
qRT-PCR	Quantitative real-time PCR
ROS	Reactive oxygen species
SOD	Superoxide dismutase
SD	Standard deviation
VPs	Versatile peroxidases
YPD	Yeast extract–peptone–dextrose
