# Supplementary material for: Overexpression of Peroxidase Gene GsPRX9 Confers Salt Tolerance in Soybean
Source: Int J Mol Sci. 2019 Jul 31;20(15):3745. doi: 10.3390/ijms20153745 (PMC6695911; doi:10.3390/ijms20153745)
Supplement: Supplementary file 1 [file ijms-20-03745-s001.zip › supplementaty/ijms-548391supplmentary.docx]

Supplementary


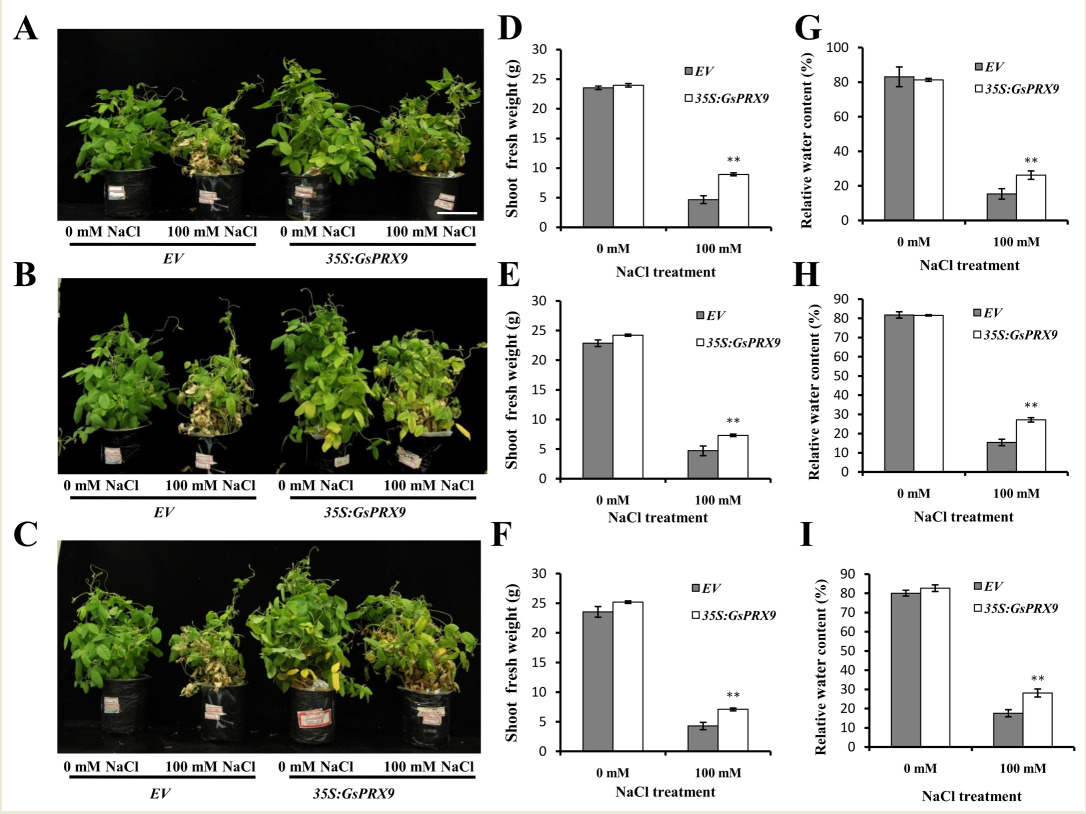


**Figure 1.** Three biological replications for salt tolerance analysis of transgenic soybean composite plants. The phenotypes (A, B, C), shoot fresh weight (D, E, F) and leaf relative water content (G, H, I) of transgenic soybean composite plants under control (0 mM NaCl) or salt stress (100 mM NaCl) were recorded 7 d after treatment. Bar = 5 cm. *EV* represents plants with the empty vector pBinGFP4, and *35S:GsPRX9* represents plants with the recombinant vector pBinGFP4*-GsPRX9*. Soybean variety of “LY01-06” was used. Data represents the mean ± standard deviation of technical repeats and each repeat has at least ten independent plants for each genotype (*n* = 10). ^∗∗^ represents significant difference between *EV* and *35S:GsPRX9* under the same condition at 0.01 level by Student’s *t*-test.


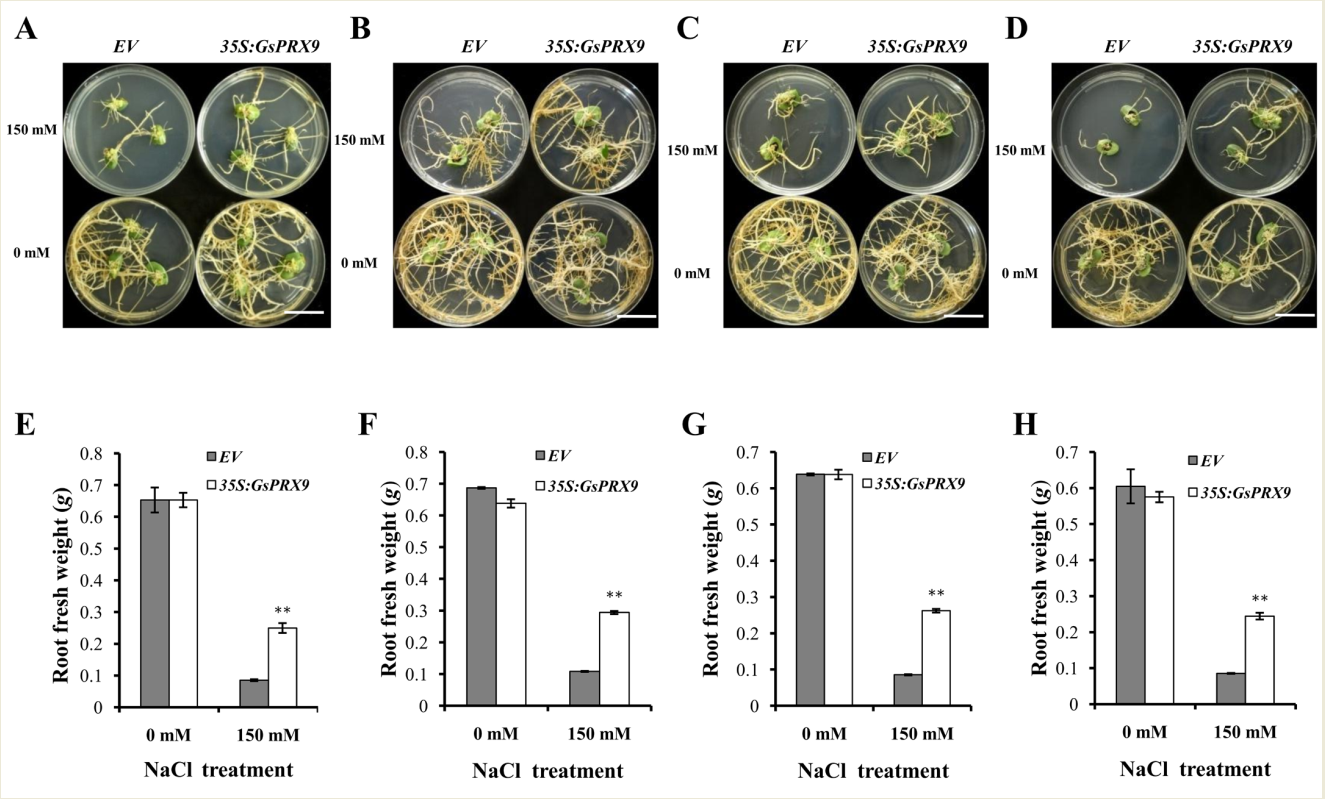


**Figure S2.** Four biological replications for salt tolerance analysis of transgenic soybean cotyledon hairy roots. The phenotypes (A, B, C, D) and root fresh weight (E, F, G, H) of transgenic soybean hairy roots under control (0 mM NaCl) or salt stress (150 mM NaCl) were recorded 14 d after treatment. Bar = 1 cm. *EV* represents roots with the empty vector pBinGFP4, and *35S:GsPRX9* represents roots with the recombinant vector pBinGFP4*-GsPRX9*. Soybean variety of “Tianlong1” was used. Data represents the mean ± standard deviation of technical repeats and each repeat has at least three independent roots for each genotype (*n* ≥ 3). ^∗∗^ represents significant difference between *EV* and *35S:GsPRX9* under same condition at 0.01 level by Student’s *t*-test.


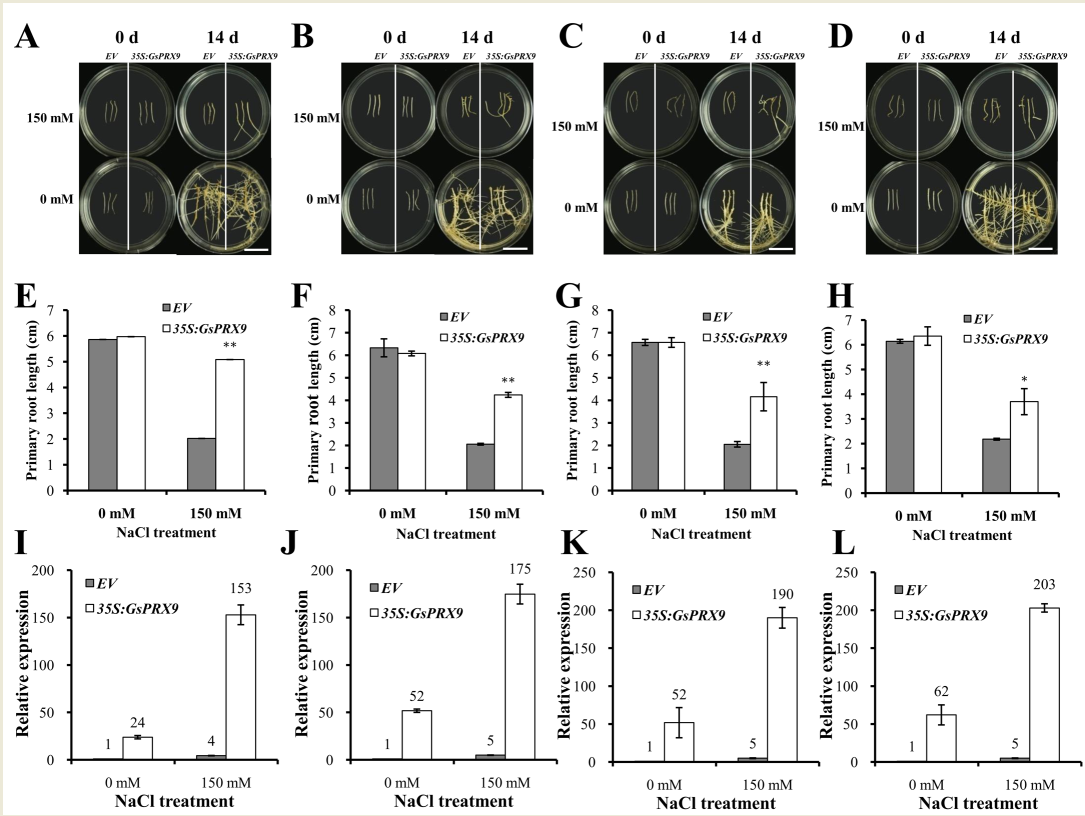


**Figure S3.** Four biological replications for salt tolerance analysis of re-generated transgenic soybean hairy roots. The phenotypes (A, B, C, D) and primary root length (E, F, G, H) of re-generated transgenic soybean cotyledon hairy roots under control (0 mM NaCl) or salt stress (150 mM NaCl) were recorded 14 d after treatment. Bar = 1 cm. (I, J, K, L) The relative expression level of *GsPRX9* in re-generated transgenic soybean hairy roots at 24 h after 0 or 150 mM NaCl treatment. *EV* represents plants with the empty vector pBinGFP4, and *35S:GsPRX9* represents plants with the recombinant vector pBinGFP4*-GsPRX9*. Soybean variety of “Tianlong1” was used. Data represents the mean ± standard deviation of technical repeats and each repeat has at least three independent roots for each genotype (*n* ≥ 3). ^∗∗^ represents significant difference between *EV* and *35S:GsPRX9* under same condition at 0.01 level by Student’s *t*-test.


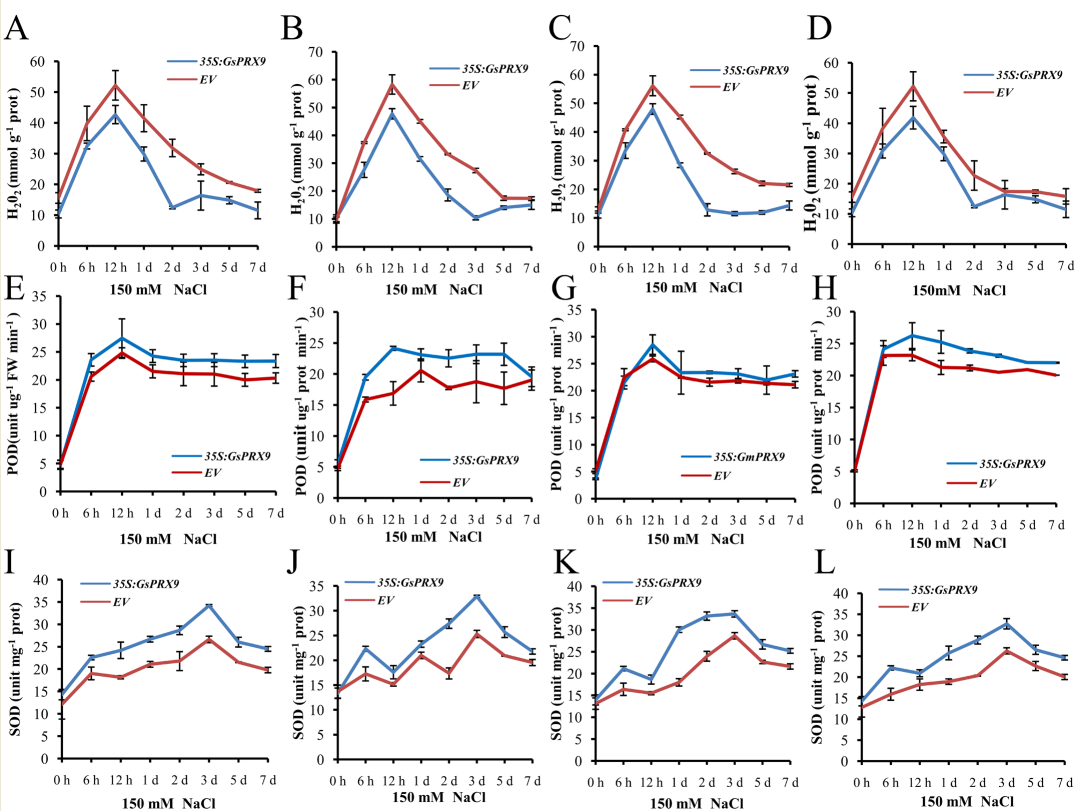


**Figure 4.** Four biological replications for the H_2_O_2_ content (A, B, C, D), POD activity (E, F, G, H) and SOD activity (I, J, K, L) in transgenic soybean hairy roots under 150 mM NaCl treatment. *EV* represents soybean hairy roots with the empty vector pBinGFP4, and *35S:GsPRX9* represents roots with the recombinant vector pBinGFP4*-GsPRX9*. Data represents the mean ± standard deviation of three technical replications.
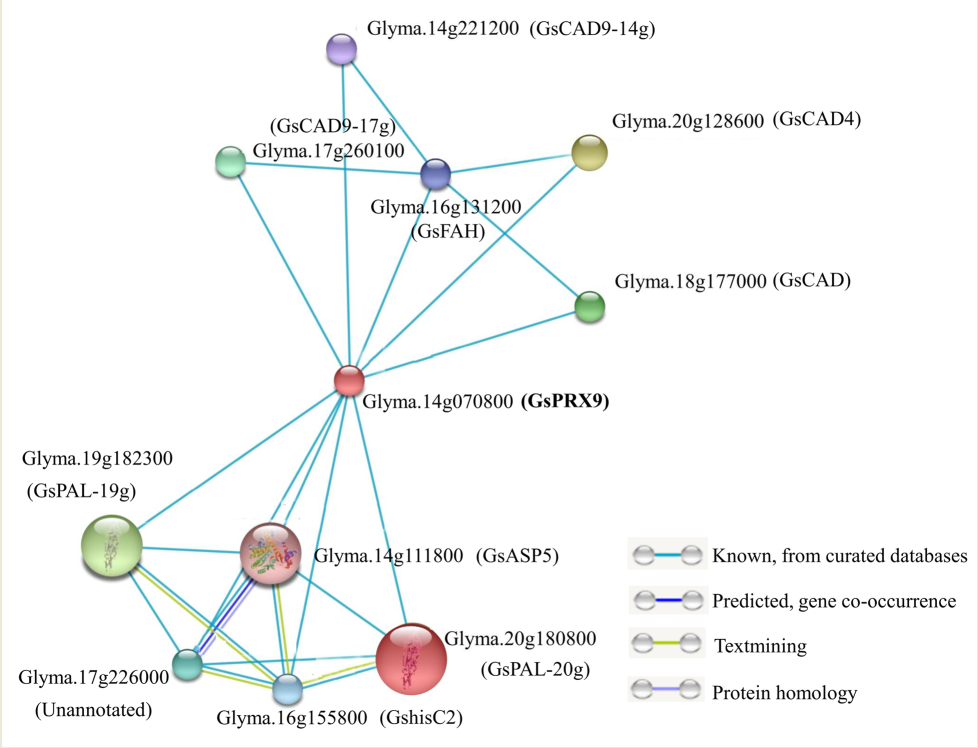
**Figure S5.T**he predicted protein-protein interaction network of GsPRX9. Figure S6. The Primer-BLAST of nine pairs of primers for qRT-PCR in this study. Figure S7. The amplicon specificities of nine pairs of primers for qRT-PCR in this study.

A. *GmUKN1*


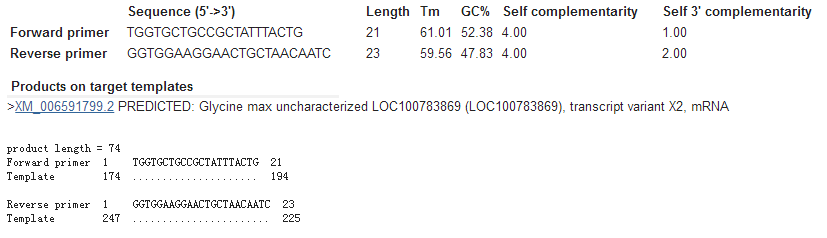


B. *GsPRX9*


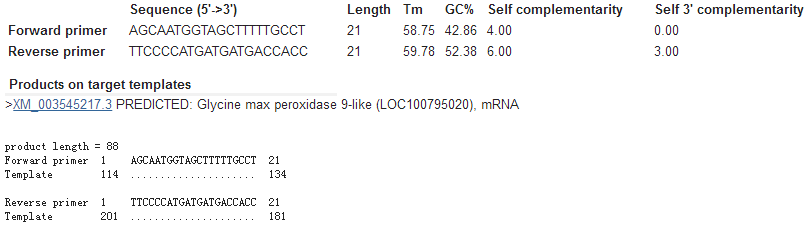


C. *GsCAD9-14g*


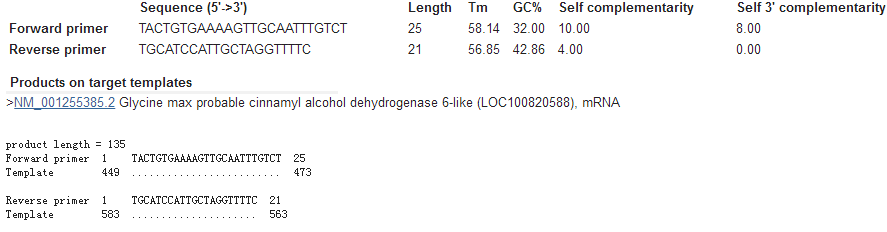


D. *GsFAH*


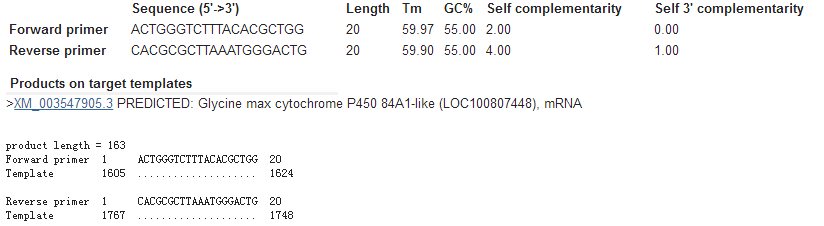


E. *GsCAD9-17g*


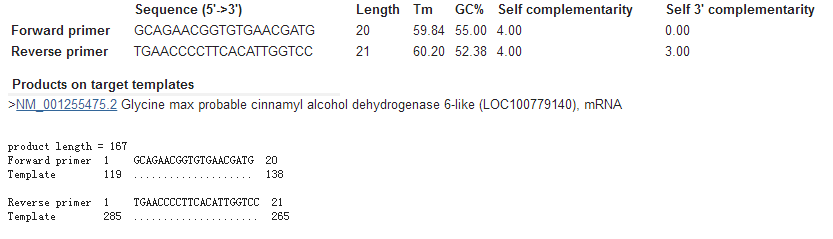


F. *GsCAD*


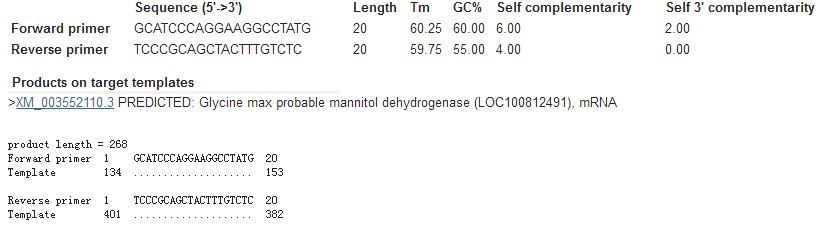


G. *GsPAL-19g*


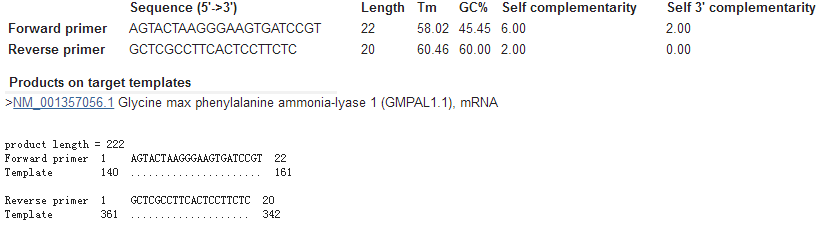


H. *GsCAD4*


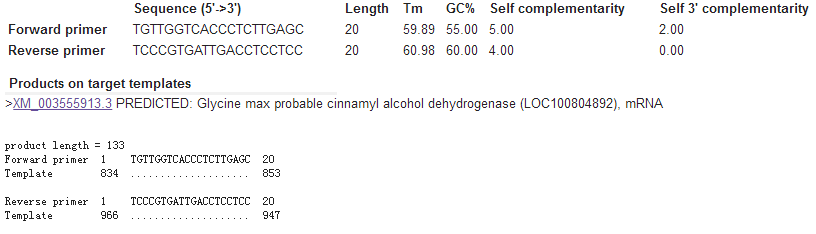


I. *GsPAL-20g*

**
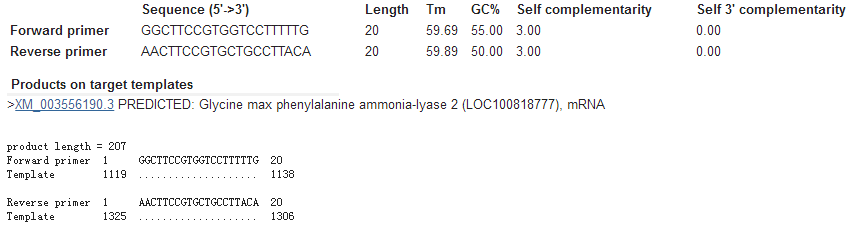
 Figure 6.** The Primer-BLAST of nine pairs of primers for qRT-PCR in this study.

A. *GmUKN1*


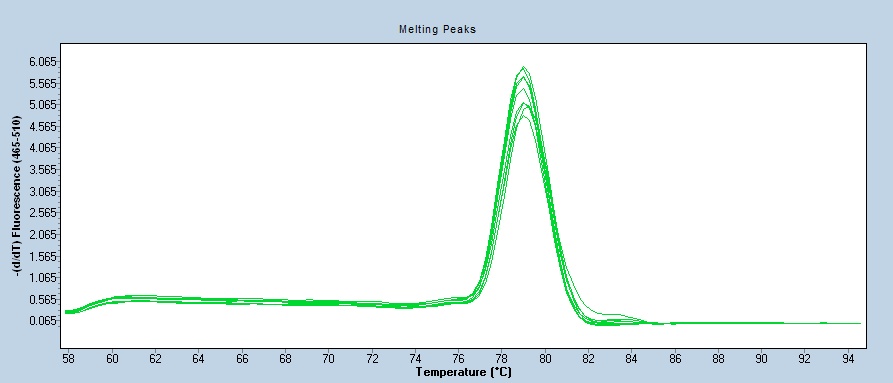


B.*GsPRX9*


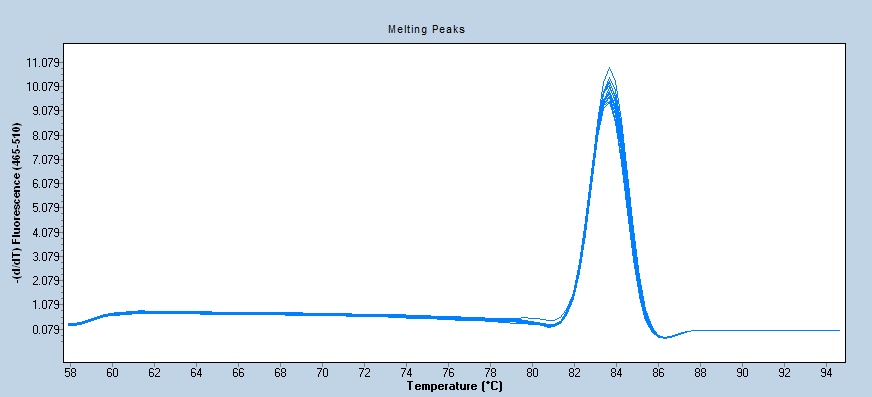


C.*GsCAD9-14g*


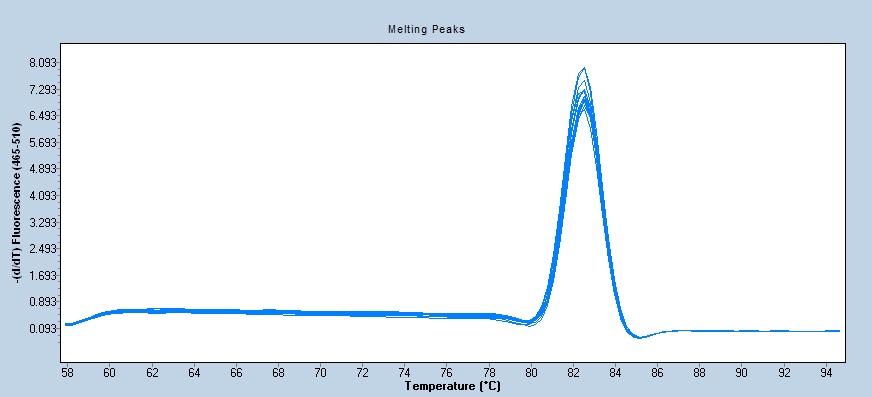


D.*GsFAH*


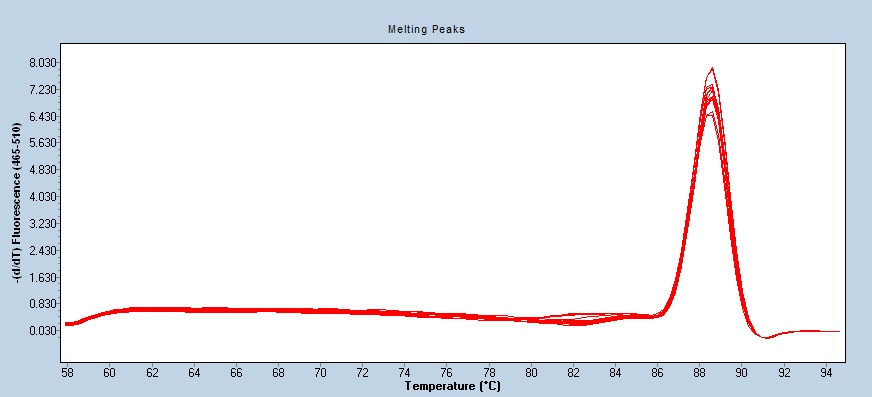


E.*GsCAD9-17g*


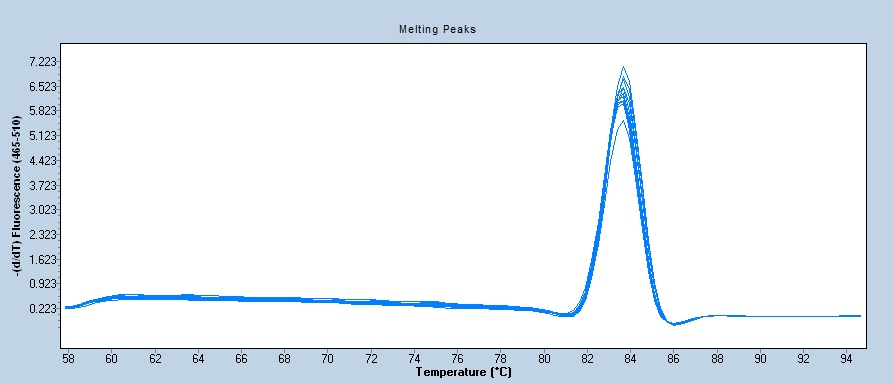


F.*GsCAD*


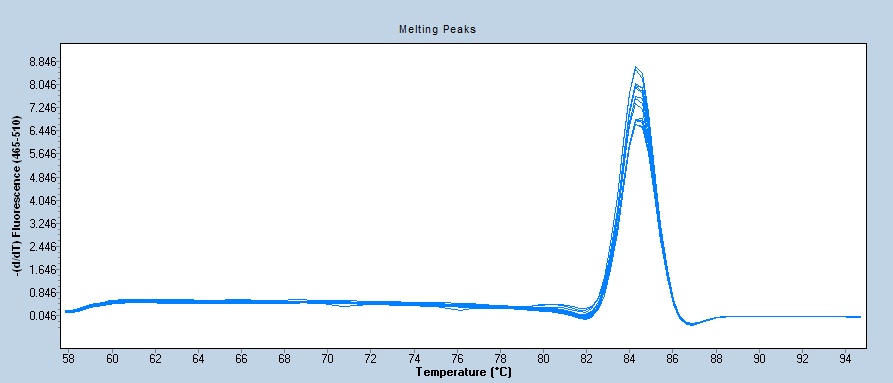


G.*GsPAL-19g*


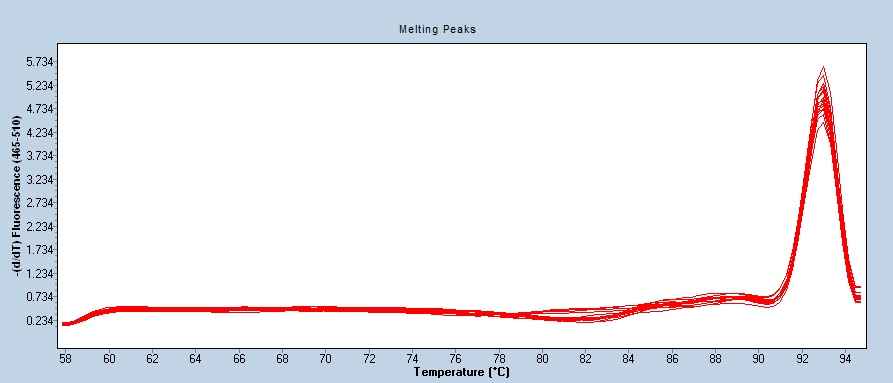


H.*GsCAD4*


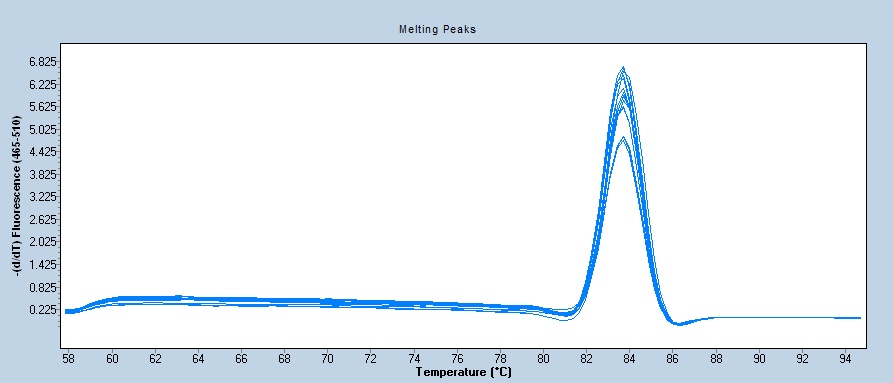


I.*GsPAL-20g*

**
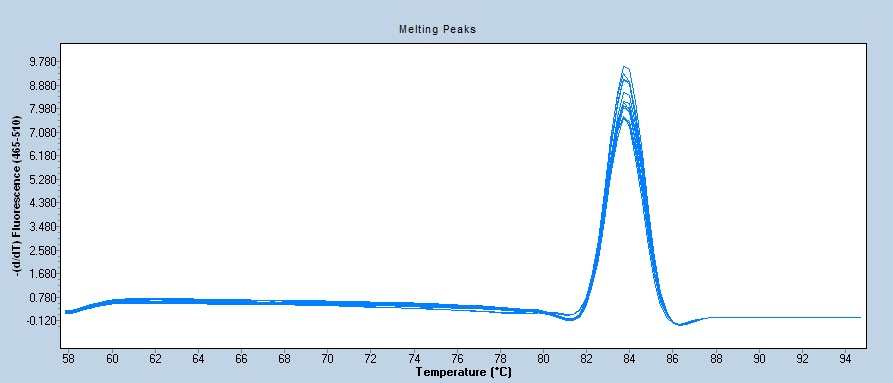
 Figure 7.** The amplicon specificities of nine pairs of primers for qRT-PCR in this study.
