# Supplementary material for: Overexpression of Peroxidase Gene GsPRX9 Confers Salt Tolerance in Soybean
Source: Int J Mol Sci. 2019 Jul 31;20(15):3745. doi: 10.3390/ijms20153745 (PMC6695911; doi:10.3390/ijms20153745)
Supplement: Supplementary file 1 [file ijms-20-03745-s001.zip › supplementaty/Table S1.docx]

**Table 1. The 159 seed sequences of peroxidases protein superfamily.**

>PER57_ARATH/40-277

VRNLVRQRFGVTPTVTAALLRMHFHDCFVKGCDASLLIDSTNSEKTAGPNGSVREFDLID

RIKAQLEAACPSTVSCADIVTLATRDSVALAGGPSYSIPTGRRDGRVSNNLDVTLPGPTI

SVSGAVSLFTNKGMNTFDAVALLGAHTVGQGNCGLFSDRITSFQGTGRPDPSMDPALVTS

LRNTCRNSATAALDQSSPLRFDNQFFKQIRKRRGVLQVDQRLASDPQTRGIVARYANN

>A2Q4C1_MEDTR/38-275

VRQVVERSFNQDRSMTAALLRMHFHDCFVRGCDASILIDSKKGNESEKAARANLTVRGYN

LIDEIKRILENACPSTVSCADIISLATRDSVVLAGGPSYNVPTGRRDGLVSTVNDVHLPG

PESSISQTLQAFKSKGMTLEEMVTLLGAHTVGFAHCSFIGKRLGSNDSSMDPNLRKRLVQ

WCGVEGKDPLVFLDQNTSFVFDHQFYNQILLGRGVLTIDQNLALDSISKGVVTGFARN

>PER44_ARATH/38-273

VASVVANRFRSDKSITAAFLRMQFHDCFVRGCDASLLIDPRPGRPSEKSTGPNASVRGYE

IIDEAKRQLEAACPRTVSCADIVTLATRDSVALAGGPRFSVPTGRRDGLRSNPNDVNLPG

PTIPVSASIQLFAAQGMNTNDMVTLIGGGHSVGVAHCSLFQDRLSDRAMEPSLKSSLRRK

CSSPNDPTTFLDQKTSFTVDNAIYGEIRRQRGILRIDQNLGLDRSTSGIVSGYASS

>P93546_SPIOL/45-290

IYNVVKQKIKADPDTVSDLVRVSFHDCFVRGCDGSIFLDGANAEKSAPVNKGLGGLKAVD

DIKAAVEKVCPGVVSCTDVLVIGARAAISLAGGKWFEVETGRRDGFVSRKNEAQASIPPP

TMPVPQAIQLFASKGLNKDDFVVLLGGHTVGTAHCHSFRERLYNFRNTKKPDSTISPTLL

QLLQKTCPRNSQTDNETFLDQTPNSHFKIDNGYYKQILAHNGVMEIDQNLALYPSTRCLV

TGLAHN

>A2YZV2_ORYSI/47-293

VQGIVRARFARDAPIVAYLLRMQFHECAVNGCDGGLLIDGPGTEKTASPNLSVKGYDLIA

DIKAELERRCPGVVSCSDIQILATRDAVVLAGGQPYAVRTGRRDRRQSRASDVVLPAPDS

TAAQTVAYFGKLGLSAFDAVLLLGAHTVGATHCGVIKDSRLYRYGGRAGATDPALDPYYA

FVYKTWVCPNAAASDGNVVFLDDQWSALRVDSNYYKQLQRRRGVLPCDQNLYGDGSTRWI

VDLLANS

>A2ZAC8_ORYSI/61-305

IHDAVQARLAWDKRMVAGLLHLIFHDCFVAGCDASILLDGPNTEKTAPQNNGIFGYDLID

DIKDTLEKACPGVVSCADIIVAATRDAVGMCGGPRYEVQLGRLDGTVSQAWMAADLPGPD

VDIPTAIDMFAKKGLNSFDMAILMGAHTVGVTHCSVIKDRLYNFNGTGEADPSMDPIYVW

ILTTFACPKSQAFDNIVYLDDPSSILTVDKSYYSQILHRRGVLAVDQKLGDHAATAWMVN

FLGTT

>A2XCM0_ORYSI/43-279

VSTVIERKFKEDPTTSALLLRLLFHDCFANGCDASILIDPLSNQSAEKEAGPNISVKGYD

IIDEIKTELEKECPQVVSCADIVALSTRDSVRLAGGPNYDVPTGRRDSLVSNREEGDSLP

GPDIAVPKLMAQFSEKGFSADEMVVLLAGGHSIGKAKCFFIEVDAAPIDPTYRSNITAFC

DGKDGDKGAVPLDPITPDVVDPNYFELVMDKKMPLTIDRLMGMDARTKPIVESMGKK

>PER60_ARATH/44-289

VSKVVGEAFIKDSSIAPAMIRLYFHDCFSNGCDASLLLDGSNSEKKASPNLSVRGYEVID

DIKSAVEKECDRVVSCADIIALATRDLVTLASGGKTRYEIPTGRLDGKISSALLVDLPSP

KMTVAETAAKFDQRKLSLNDMVLLLGGHTIGVTHCSFIMDRLYNFQNTQKPDPSMDPKLV

EELSAKCPKSSSTDGIISLDQNATSSNTMDVSFYKEIKVSRGVLHIDQKLAIDDLTSKMV

TDIANG

>A5BTV8_VITVI/77-327

VRSQVXFYWNELKDGSIAPKLLRLLYSDCFVNGCDASILLDGPNSEKTASQNRGLGGFAL

IDKIKTVLESRKECKGVVSCADILNLATRDAVHLAGAPSYPVLTGRKDGLESNAASVDLP

SPSISWESALAYFKSKGLDVLDLGTLLGAHTLGRTHCSYIEBRLYNFNGTNKPDPSMDTS

FLAEMKKKCPQRVKKGQXDPLVFLNPESGSSHNFTNSYYSRILSHKAVLGVDQQLLFGBD

TEQITEEFAAG

>A2YCW1_ORYSI/47-300

VRDVVTSEIGLDRTIAAGIIRIFFHDCFVTGCDASILLDETPSGDVPEKESSANGFTLHG

LRTLDVAKSTVESMCPRTVSCADILAFAARDAAVAAGIPFYDVAAGRMDGLRSNMDDLPG

NMPTPSHQVPRMSELFVKRGLSQEDLVVLSGAHSIGGAHCFMFSNRIYGFSQGADIDPAL

EPAFAEKLRKVCPPRKDGDDPEQSPKVSFDGRTSEKLDNVYYSELLASRGLMTSDDALIK

DPETKTTVDLFAGD

>PER47_ARATH/53-289

VKNSVNNALQADPTLAAGLIRMLFHDCFIEGCDASILLDSTKDNTAEKDSPANLSLRGYE

IIDDAKEKIENRCPGVVSCADIVAMAARDAVFWAGGPYYDIPKGRFDGKRSKIEDTRNLP

SPFLNASQLIQTFGQRGFTPQDVVALSGAHTLGVARCSSFKARLTVPDSSLDSTFANTLS

KTCSAGDNAEQPFDATRNDFDNAYFNALQMKSGVLFSDQTLFNTPRTRNLVNGYALN

>B8BA60_ORYSI/43-280

VRSVVSQALMGDPSLAASLLRLHFHDCFVQGCDASVLLDSTPDNTAEKDALANKSLRGFE

VIDRIKDALESRCPGVVSCADVLALAARDAVIMAGGPYYGVATGRRDGTRSSAADTVALP

PPFLNATALIQLFGTHGFTAQDMVALSGGHTLGRAHCANFKNRVATEAATLDAALASSLG

STCAAGGDAATATFDRTSNVFDGVYFRELQQRRGLLTSDQTLFESPETKRLVNMFAMN

>PER18_ARATH/47-293

VRNTVRSASSSDPSVLGKLLRLIFHDCFVQGCDGSVLIRGNGTERSDPGNASLGGFAVIE

SVKNILEIFCPGTVSCADILVLAARDAVEALGGPVVPIPTGRRDGRVSMAANVRPNIIDT

DFTVDKMINIFSSKGLSVHDLVVLSGAHTIGAAHCNTFNSRFKLDPKGNLELIDASLDNS

YAQTLVNKCSSSLDPTTTVVDNDPETSSTFDNQYYKNLLAHKGLFQTDSALMEDDRTRKI

VEILAND

>Q7XT82_ORYSJ/180-476

LAKFLRAVLPERGAGGEGCRQGCDASVMIEGSGTERTDPANLSLGGFNVIDAAKRLLEAV

CPVTVSCSDILVLAARDAVTFVSMMALCDFSFLVATIHSKTGGPLVPVSLGRLDGLVSLA

SNVRANIIDTGFSVDAMARSFSAKGLTLDDLVTLSGTHTHALECWRINCDFHFSPGLTVS

TRLSIAGVAHWRAGGHTIGSAHCTTFGERFRVDANGSTVPADAAMNADYAGGLIRACSAV

NNTVSSTAAVDCDEGSASRFDNAYFANLLAGRGLLRTDAVLVQNATTRATVEAFARS

>PER19_ARATH/58-309

VGSVTSQRFKEVPISAPATIRLFFHDCFVEGCDGSILIETKKGSKKLAEREAYENKELRE

EGFDSIIKAKALVESHCPSLVSCSDILAIAARDFIHLAGGPYYQVKKGRWDGKRSTAKNV

PPNIPRSNSTVDQLIKLFASKGLTVEELVVLSGSHTIGFAHCKNFLGRLYDYKGTKRPDP

SLDQRLLKELRMSCPFSGGSSGVVLPLDATTPFVFDNGYFTGLGTNMGLLGSDQALFLDP

RTKPIALEMARD

>PER55_ARATH/48-294

VKQAVTTKFKQTVTTAPATLRMFFHDCFVEGCDASVFIASENEDAEKDADDNKSLAGDGF

DTVIKAKTAVESQCPGVVSCADILALAARDVVVLVGGPEFKVELGRRDGLVSKASRVTGK

LPEPGLDVRGLVQIFASNGLSLTDMIALSGAHTIGSSHCNRFANRLHNFSTFMPVDPTMD

PVYAQQLIQACSDPNPDAVVDIDLTSRDTFDNSYYQNLVARKGLFTSDQALFNDLSSQAT

VVRFANN

>A2XM89_ORYSI/42-273

VLGVVKDKMQATIRTIGSTVRLFFHDCFVDRDAPDNLSLAFEGFETVRSAKAAVEAACPD

QVSCTDVLAIATRDAIALSGGPFFPVELGRLDGMRSSASNVAGKLPQPNNTLSELVAIFK

SNGLNMSDMVALSAAHSVGLAHCSKFSDRLYRYNPPSQPTDPTLNEKYAAFLKGKCPDGG

PDMMVLMDQATPALFDNQYYRNLQDGGGLLASDELLYTDNRTRPTVDSLAAS

>Q5Z8H9_ORYSJ/28-264

VRGSVQRSMQQSPIAAPATLRLFFHDCAVRGCDASIMIINPNGDDEWRNPDDQTLKPEGF

TTVIAAKAAVDSDPQCRNRVSCADILALATRDSIFLSGGPNYAVELGRFDGRVSTRNSVN

LPHGNFNLDQLTGYFGSLGLSPTDMVALSGGHTIGAASCNFFGYRLGGDPTMDPNFAAML

RGSCGSSGFAFLDAATPLRFDNAFYQNLRAGRGLLGSDQTLYSDPRSRGLVDRYAAN

>A2YGK4_ORYSI/51-299

VRGAVTQKLKETFNAAPGTLRLFFHDCFVRGCDASVLIAGPDDEHSAGADTTLSPDALDL

ITRAKAAVDADAQCANKVSCADILALAARDVVSQAGGPYYQVELGRLDGKVGTRAVVKHS

LPGAAFDLDQLNKLFATNGLTQTDMIALSGGHTIGVTHCDKFVRRLYQFKGAAPQYSPPM

NLAFLRQMRQTCPLSYSPTTVAMLDAVSPNKFDNGYFQTLQQLKGLLASDQVLFADRRSR

ATVNYFAAN

>Q8H225_SETFA/40-272

VRAEVKKAVRANTGVGAGLIRMHFHDCSVRGCDASVMLMAPNGGDESHSGADRHCRQTPW

TPSTRPRRPWRAPRVRRKVSCADILAMAARDVVSLLGGPNYAVELGRLDGKSFNRAIVKH

VLPGPGFNLDQLNSLFAQNGLTQTDMIALSGCHTIGVTHCDKFIRRIYTFKQHLPWNPPM

NLEYCGRCVGCAPSTTARRRSRCWTRPRPGSSTTPTSTTSATTRACSPRTRTS

>A3AUM5_ORYSJ/50-276

VADVVAAKQRANPSTAAGTLRLFFHDCFVGGCEALGPRLPALRRPVALEVACPGTVSCAD

ILALAARDLVGILGGPRFPVALGRRDARRSDARDVEGNLPRTNMSARAMAVLFARKGFTP

RELVALAGAHTVGFSHCGEFAHRLYSFRSADGYDPSLNPAFARALQSSCANYRSDPTISI

FNDIMTPGKFDEVYFKNLPRGLGLLASDAALWEYPATRVFVQRYADN

>A5BQ28_VITVI/38-287

MQDAITSKQINSPTTAAGTLRLFFHDCMVDGCDASVLISSNAFNTAERDADINLSLPGDA

FDLIVRAKTSLELTCPGIVSCADILALATRDLVTMVGGPYYDVQLGRKDGLVSQASRVEG

NLPRANMTMDQLIAIFAAKGFSIQEMVALSGGHTIGFSHCKEFSNRIFNYSSTSDIDPAF

HPKFAQALRNVCANYQRDTAMSAFNDVMTPNKFDNMYYQNLPRGLGLLSSDNVLVTDPRT

KPFVELYATN

>M4FGE7_BRARP/42-287

VRETVTTTQGPQGRTAAGILRLFFHDCFLEGCDASVLIAKNALNKSERDDELNHSLTEET

FDIVTRIKAALEESCPGVVSCADILAQSTHDVVTMIGGPSYEVKLGRKDGFESKAHKVRE

NLPLPNHTVHDMMSLFQKKGFTLKEMVALSGAHTIGISHCKDFISRVIGPQPDPDIEARY

AEVLKSLCKDYTVNETRGSFLDPVTPDKFDNMYYKNLEKGMGLLASDHILFKDNSTRPFV

ELYAND

>PER64_ARATH/39-284

VTNAVKKAMSNDQTVPAALLRMHFHDCFVRGCDGSVLLDSKGKNKAEKDGPPNISLHAFY

VIDNAKKALEEQCPGIVSCADILSLAARDAVALSGGPTWAVPKGRKDGRISKAIETRQLP

APTFNISQLRQNFGQRGLSMHDLVALSGGHTLGFAHCSSFQNRLHKFNTQKEVDPTLNPS

FAARLEGVCPAHNTVKNAGSNMDGTVTSFDNIYYKMLIQGKSLFSSDESLLAVPSTKKLV

AKYANS

>A2WNB5_ORYSI/47-289

VRSVTWAQVAANPALPGRLLRLHFHDCFVQGCDASILLDNAGSEKTAGPNLSVGGYEVID

AIKTQLEQACPGVVSCADIVALAARDAVSYQFKASLWQVETGRRDGPVSLASNTGALPSP

FAGFSTLLQSFANRGLNLTDLVALSGAHTIGKASCSSVTPRLYQGNTTSLDPLLDSAYAK

ALMSSCPNPSPSSSTIDLDVATPLKFDSGYYANLQKKQGALASDAALTQNAAAAQMVADL

TNP

>Q40486_TOBAC/47-294

VRDITWSKAKNDATLSAKLLRLHYHDCFVRGCDASILLDKVGTDQSEKEARPNLSLGGFD

VIDDIKRQVEEKCPEIVSCADILALAARDAVSFPFKKSLWDVATGRKDGNVSFGSEVNGN

LPSPFSDFATLQQLFAKKGLNVNDLVALSGAHTIGVAHCGAFSRRLFNFTGKGDMDPSLN

PTYVESLKQLCPNPANPATTVEMDPQSSTSFDSNYFNILTQNKGLFQSDAVLLTDKKSAK

VVKQLQKT

>Q67Z30_ARATH/62-313

VRQIVWKKVEANRSLAPKLLRVHYHDCFVRGCDASLLLDSVAGKAVSEKEARPNLSLSGF

EIIDEIKYILEKRCPNTVSCADILTLAARDAVSYEFERPLWNVFTGRVDGRVSLATEAAR

DLPSAGANFTTLQKLFAESDLDVVDLVALSGAHTIGIAHCGVFGRRLLNFTGKGDTDPSL

NPSYASFLKSECSDKSLRLNPSAVVGMDPTGPLAFDSGYFVSLLKNKGLFTSDAALLTDP

SAAHIASVFQNS

>Q8L4E6_ORYSJ/45-289

VRSELKGIFSNDTTLRAGLLRLHFHDCFVRGCDASLMLNSHNATAEKDADPNLTVRGYEA

IEAVKAKVEATCPLVVSCADIMAMAARDAVYFSDGPEYEVETGRRDGNVSNMAEALTNLP

PSDGNVTVMTQYFAVKNLTMKDMVVLSAAHTIGVAHCTSFSKRLYNFTGAGDQDPSLDPA

FAKQLAAVCKPGNVASVEPLDALTPVKFDNGYYKSLAAHQALLGSDAGLIDDSLTGAYVR

LMTND

>A2Y668_ORYSI/53-310

VLGEMRMILEEDPTLAPSLLRMHYHDCFVQGCDGSIMLRSRSGKGERDATPNRSMRGYDA

INRIKARLETVCPLTVSCADIIAMAARDAVYLSKGPWYDVETGRRDGDVSVAEYAENDLA

PPDSNIVDVKTFFSVKSLNAKDIAVLFGCHSIGTSHCGAFQKRLYNFTGRMDQDPSLDAG

YAAKLKKLCPPGHGHDHDHDGHGGAGGAAKVPMDPGSGFTFDLSYYRHVLATGGLFQSDG

SLRDDPVTRGYVEKLANA

>Q43158_SPIPO/41-292

ITEEIDRAIRVAPSIGGPLLRLFFHDCFVRGCDASLLLNATSSSNPTEKDAPPNQFLRGF

ALIDRIKARLERACPSTVSCADILALIARDVVHADQGPFWQVPTGRRDGFVSIASEATQL

LPAFSANISTLKSQFNDVGLSAKDLVLLSGGHTIGNAHCFTFTTRLYNFSGRGDNSDTDP

SLERNYLAKLRAKCAQDGSDALKLVEMDPGSFTTFDNSYFKLVAKRRGLFQSDAALLDDA

DTRSHVIHLAES

>A2XXV5_ORYSI/43-289

VRDEMTKIISRVPSLAGPLLRMHFHDCFVNGCDGSILLDSTPGSPSEKESIPNLSLRGFG

TIDRVKAKLEQACPGVVSCADILALVARDVVFLTKGPHWEVPTGRRDGTRSVKDDAVNNL

PPPFFDATRNLYQFFIPKGLDAKDQVVLLGGHTLGTSHCSSFASRLYNFSGMMMADPTLD

KYYVPRLKSKCQPGDKTTLVEMDPGSFRTFDTSYYRHIARGRALFTSDETLMLDPFTRGY

ILRQAGV

>I1Q7D6_ORYGL/56-304

VRREMEEILRVAPTLAGPLLRLHFHDCFVRGCDASVLIDSTAGNVAEKDAKPNLTLRGFG

AVQRVKDKLNAACPATVSCADVLALMARDAVVLANGPSWPVSLGRRDGRLSIANDTNQLP

PPTANFTQLSQMFAAKGLDAKDLVVLSGGHTLGTAHCALFSDRLYNFTGLVNDGDVDPAL

DAAYMAKLKAKCRSLSDNTTLSEMDPGSFLTFDASYYRLVAKRRGIFHSDSALLTDPVTR

AYVERQATG

>Q8L4Z9_ORYSJ/62-309

VFRETARIIRASPDLAAALLRLHYHDCFVQGCDASVLLDSTRANAAERDSDPNKSLRGFD

SVARVKAKLEAACPATVSCADLLALMARDAVVLAKGPYWHVPLGRRDGRSSTAASCGGQL

PPLCGNVSRMVDSFAAKGLDVKDLVVLSAAHTLGKAHCPNFADRLYGPGADPPLKLDGAY

ADRLRKQCKEGAPPYDGNVTAEMDPGSFTRFDSSYFRQVVRRRALLRSDACLMDHPFTSA

YIRLAATG

>A2YHB9_ORYSI/46-289

VYKEMTSILAKSPELAGPVLRLFSVDCFVGGCEGSILLDSTPGNKAEKDSPLNKGVKGYE

VVDAIKAKLDAACPGIVSCADTLALAARDVVRLTKGPYIPLPTGRRDGNSSNAADVAANS

PAPDATVNDLLTIFAKFNFTAKDLAVLSGAHTIGKAHCSAFSTRLYSNSSSNGGPTLDAN

YSTALRGQCKVGDVDTLVDSNPQPDTFDTDYYKQVAAQRGLLATDAALLLNADTKAYVLR

QANA

>Q8GVP1_ORYSJ/42-299

VRQEVASVLSVAPYLAGALLRLHFHDCFVRGCDGSILLDSVAGGAVDAEKEAETSAGLRG

FDVIDSIKEKLEQACPGTVSCADILALAARDAVHWSNGPFWPVPTGRLDGKISNAAETVD

LPPPNSGMAQLQAAFAHKNLTAKDLVVLSGAHTIGFSHCQPFHDRLYNYTGGNRLNDVDP

ELDPAYLNELRSKCGAAASATANADNPGVMVEISPKRSPKFDTGYYTQVARRRGLFRSDA

VLLDDDFTGAYVKKHATG

>A2XIK7_ORYSI/55-312

VRKVVAAAVHDDPTTTAPLLRLHFHDCFVRGCEGSVLINSTKKNTAEKDAKPNHTLDAYD

VIDAIKEKLEHKCPATVSCADILAIAARDAVSLATKAVRQGRWSKDGNLYEVETGRRDGR

VSSAKEAVTYLPDSFDGIRRLITRFASKGLSLKDLAVLSGAHALGNTHCPSIAKRLRNFT

AHHNTDPTLDATYAAGLRRQCRSAKDNTTQLEMVPGSSTTFDATYYGLVAERKGMFHSDE

ALLRNDVTRGLVYEYMRS

>Q9ZNZ6_SOYBN/67-314

VLKFVHDHIHNAPSLAAALIRMHFHDCFVRGCDASVLLNSTTNQAEKNAPPNLTVRGFDF

IDRIKSLVEAECPGVVSCADILTLSARDTIVATGGPFWKVPTGRRDGVISNLTEARDNIP

APSSNFTTLQTLFANQGLDLKDLVLLSGAHTIGIAHCSSLSNRLFNFTGKGDQDPSLDSE

YAANLKAFKCTDLNKLNTTKIEMDPGSRKTFDLSYYSHVIKRRGLFESDAALLTNSVTKA

QIIELLEG

>Q9ZTW8_SOYBN/43-266

LKYVVEHIPQRSITSSCSHKGLHFHDCFVNGCDGSVLVELNTRGIKLKRNAIPNLTISEA

LGFIEAIKRLVEAECPGGSLLVLISWLWTARDSIHATGGPYWNVPTGRRDGFISRAADPL

RQPSCSFSQPHYLTQPNTLWAMLDLNANDLVLLVGAHTIGIAHCSSISTRLYNFTGKGGH

RPNNRQWICKKSQDLQSVRTLMITHLLRWTLEVVIHLILDITNK

>PER5_VITVI/44-292

VKDEVRKGFIRDSGVAPGLVRMHFHDCFVRGCDGSVLIDSTPSNTAEKDSPANNPSLRGF

EVIDSAKARLEAVCKGVVSCADIVAFAARDSVEITGGLGYDVPAGRRDGRISLASEASTN

LPPPTFTVDQLTQFFSNKGLTQDEMVTLSGAHTIGRSHCSSFSNRLYNFNGTSGQDPTLD

PQYAASLKTQCPQGSTNTNLVVPMNPSSPSITDVGYYVDVLRNRGLFTSDQTLLTDTTTA

TQVRQNAGN

>Q5U1M3_ORYSJ/43-296

VRNAVRRAVARDPGLAAGLIRMHFHDCFVRGCDGSILINSTPGHVAEKDSVANNPSMRGF

EVVDDAKAIVEAHCPRTVSCADILAFAARDSAHLAGATVDYPVPSGRRDGRVSVSDEVLA

DNVPAPTFSLAQLVASFERKGLTADDMVTLSGAHTIGRSHCSSFTARLYNFSGEAGRTDP

AIDPAYAAELKRRCPPATDDQMDPTTVPLDPVTPASFDNQYYKNVLKHRVVLNSDQALLD

SPWTAGVVKLHSAV

>Q6AVZ3_ORYSJ/57-307

VRDTVTKAFEKAPGTPADLIRLFFHDCFVRGCDASVLLESTPGNKAERDNKANNPSLDGF

DVVDDAKDLLEKECPHTVSCADILSLVARDSAYLAGGLDFEIPTGRRDGFVSKEDEVLSN

VPHPEFGAKDLLKNFTAKGFTAEEMVTLSGAHSIGTSHCSSFTNRLYKYYGTYGTDPSMP

AAYAADMKSKCPPETAAQQDATMVQLDDVTPFKMDNQYYRNVLAGNVTFASDVALLDTPE

TAALVRLYAAG

>Q9LGT9_ORYSJ/38-257

ISNVVYGLIDADPSMAPALLRLHFHDCFVMGCDASILLDPTKANGSPEKTAIPLRGYDAV

NKIKAAVEAVCPGKVSCADILAFAARDSVTKSGGFVYPVPSGRRDGDVSSAFSVFSSIPS

PFFDADELVQSFAAKGLTVDDLVALSEPAVPDGGRLPGRELRGGAAADDGVVNNSPVSPA

TLGNQYFKNALAGRVLFTSDAALLAGRNDTAEKVRENAGD

>Q8W2X3_ORYSJ/45-283

VFDEVQKAWNADRSMPASLLRLHFHDCFVNGCDGSVLLEASDGQAEKNAQPNLSLRGYDV

VDRVKARLEATCKQTVSCADILAYAARDSVRVMTGGYKYEVPGGRPDGTVSRASMTGDLP

PPKQRNVDQLARYFTSKGLTVDDMVVLSGAHTLGVARCGTFGYRLTSDGDKGMDAAFRNA

LRKQCNYKSNNVAALDAGSEYGFDTSYYANVLANRTVLESDAALNSPRTLARVTQLRGN

>A2Y049_ORYSI/51-296

IQTIVHGAVRNDAGNGPGLIRLFFHDCFVRGCDASVLLDADPASNGTVEKMAPPNFPSLR

GFGVIDRAKRVVERRCPGVVSCADIVAFAARDASRIMGGIKFAMPAGRLDGRVSSASEAL

ANLPPGSFNLTQLVARFATKNLTADDMVTLSGAHSIGRSHCSSFSSRLYPQIDPAMNATL

GVRSRAKCAAAPGRLDRVVQLDFKTPLQLDNQYYQNVLTHEVVFTSDQSLIDRPDTAALV

AQYAGS

>Q5U1P5_ORYSJ/57-303

VKGVVAAALHRDPGVGAGLIRMLFHDCFVEGCDASVLLDPTPANPQPEKLAPPNNPSLRG

FEVIDAAKDAVEAACPGVVSCADIVAFAARDASFFLSDSRVSFDIPSGRLDGRYSNASRA

LDFLPPPTFNLGQLVANFAAKGLSVEDMVVLSGAHTIGLSHCSSFVSDRLAVASDIDPSF

AAVLRAQCPASPSSSNDPTVVQDVVTPNKLDNQYYKNVLAHRALFTSDASLLASPATAKM

VVDNANI

>B9FGW7_ORYSJ/46-310

VRKEVEKAIKHNPGVGAALVRLVFHDCWVNGCDGSVLLDKTPYSSSTEKAAANNIGLDGF

DVIDAIKSKLGAAVSCADIVVLAGRDASAILSGGRITYDVGTGRKDCVVSSAAAADAVLP

ESTFDFAQLKDNFASKGLTQGELVILSGAHSIGVAHLSSFHDRLAAATATPIDATYASAL

AADVERQKGVQRTDNPAEKNNIRDMGAAFQSAAGYDAAGVDTAAVGALDNSYYHNNLQNR

VLFKSDWVLRTDGDAAADLAEYRDN

>A2WNS0_ORYSI/48-308

VYNTVKDFLDADRSKGAALVRLLFHDCFVRGCDGSILLDNSTANPTPEKMSGANIGIAGL

DVIDAIKAKLETACPGVVSCADIVVFAGRDASRYMSNGGVSFDVPAGRLDGVVSSAADAT

NTLPDSKTGVATLISNFAKKGFTPEELVILSGAHSIGKAHCSNFDDRLTAPDSEINADYR

DNVLSKTCKSAPNPTLANNIRDIDAATLGDLASYVVPAVGGDYLDNSYYKNNKNNLVLFH

SDWALVGSNATLQHVNEYAAN

>A2Y045_ORYSI/42-308

VNSIVVNSIKANRGKGAGLVRLLFHDCFVRGCDASVLLEKSEMNMHPEKESQANIGIRGM

DVIDAIKAALEARCPNTVSCADIIAYAARDASRYLSRGGVDFPVPGGRLDGVVSRSRDAD

AFLPDSAANLTGLVRNFRRKNFTVEELVILSGAHSIGVTHCTSFAGRLTAPDAQINPGYR

NLLVSKCGGVSPTATNNHVVVNNVRDEDGAAVARAMPGFAARVRKARDYLDNSYYHNNLA

MAVTFHADWALLTGKEARGHVVEYAKN

>Q6AVZ8_ORYSJ/42-302

VRYHVAKALKANRKEGAALVRLIFHDCFVRGCDASVLLDPTAENPHTEKTAPINIGLAAF

ELIDEIKAAVEERCPGVVSCADIVIYAARDASILLSNGHVHFDVLAGRLDGRVSRAADAQ

RDLPDSTFTISELIRNFRRKNFTIEELVILSGAHAVGVGHCSSLRARLTAPPEQILPGYR

SLLAGKCAAGEDPIVPNNVRDEDPAAVAATIPSFLPKLRKFEFLDNSYYHNNLARIVTFN

SDWQLLTEKKARGHVHEYADN

>Q5U1S9_ORYSJ/45-290

VRQAVTNAFANDSGIAAGLIRLHFHDCFVRGCDASVLLTSPNNTAERDAAPNNPSLRGFQ

VIDAAKAAVEQSCARTVSCADIVAFAARDSVNLTGGVSYQVPSGRRDGNVSVAQDAIDNL

PQPTFTAAQLVASFANKSLTAEEMVVLSGAHTVGRSFCSSFLARIWNNTTPIVDTGLSPG

YAALLRALCPSNASATATTAIDVSTPATLDNNYYKLLPLNLGLFFSDNQLRVNATLGASV

SSFAAN

>Q6RFL1_MAIZE/40-299

VQQTVAAAFRNNSGVAPALIRMHFHDCFVRGCDGSVLIDTVGNLTAEKDAPPNNPSLRFF

DVVDRAKASLEAQCPGVVSCADVLAFAARDSVVLSGGLGYQVPAGRRDGRISNDTEALNN

LPPPFFNATELADRFASKNLSIEDLVVLSGAHTIGVSHCSGFAGPTDLNGPVDRLYNFSS

PDGIDPTLSKAYAFLLKSICPANTSQFFPNTTVFMDLITPERFDNKYYVGLTNNLGLFKS

DVALLTNATMKALVDSFVRS

>Q7XSU8_ORYSJ/56-288

VRSAVQAALQREIALAAGLIRIFFHDCFPQGCDASVYLSGANSEQGMPPNANSLQPRALQ

LVEDIRAKVHAACGPTVSCTDISALATRAAVVLSGGPTYPVPLGQLDSLAPAPLRLVNQL

PGPGTSSVQALIDLFGSRGMGDAADLVALSGGHTVGKSKCAFVRPVDDAFSRKMAANCSA

NPNTKQDLDVVTPITFDNGYYIALTRKQGVFTSDMALILDPQTAAIVRRFAQD

>B8ARU3_ORYSI/50-290

VRWHVTEALRRDIGIAAGLVRIFFHDCFPQGCDASVLLTGSQSELGEIPNQTLRPSALKL

IEDIRAAVHSACGAKVSCADITTLATRDAIVASGGPYFDVPLGRRDGLAPASSDKVGLLP

APFFDVPTLIQAFKDRNLDKTDLVALSGAHTIGLGHCGSFNDRFDGSKPIMDPVLVKKLQ

AKCAKDVPVNSVTQELDVRTPNAFDNKYYFDLIAKQGIFKSDQGLIEDAQTNRTAVRFAL

N

>Q5W5I2_PICAB/49-295

VRQRMGAYLSADITQAAGLLRLHFHDCFVQGCDGSVLLNSTSGEQTTPPNLSLRAQAFKI

INDIKQHVEAACSGIVSCADILALAARDSVAMAGGPFYPIPFGRRDSLTFANLSTTLANL

PSPTSNVTVLISVLGPKGLTFTDLVALSGGHTIGRSNCSSFQNRLYNSTTGISMQDSTLD

QNFAKNLYLTCPTNTSVNTTNLDILTPNVFDNKYYVDLLNEQTLFTSDQSLYTDTRTRDI

VKSFALN

>Q6PQF2_EUPCH/48-291

IQKELKKLFKKDVEQAAGLLRLHFHDCFVLGCDGSVLLNGSAGGPSEQSELPNLSLRKQA

FKIVNDLRALVHKECGPVVSCSDIVAIAARDSVVLTGGPKYDVPLGRRDGVKFAEVNATF

EHLVGPTANVTTILAKLARKGLDTTDAVSLSGGHTIGIGHCTSFTERLYPSQDPTLDKTF

ANNLKRTCPNVNTENSTFLDLRTPNEFDNRYYVDLMNRQGLFTSDQDLYTDKRTRQIVID

FAVN

>Q9XFI8_SOYBN/51-288

VRKHLKKVFKDDNGQAPALLRIFFHDCFVQGCDGSLLLDGSPSERDQPANGGIRTEALQT

IDDIRAIIHKECGRIVSCADITVLAARDSVFLTGGPDYAVPLGRRDGLSFSTSGTSDLPK

PFNTTGVTLDAFAAKNFDVTDVVALSGAHTFGRAHCGTFFNRLSPLDPNMDKTLAKQLQS

TCPDANSGNTANLDIRTPTVFDNKYYLDLMNRQGVFTSDQDLLNDKRTKGLVNAFALN

>A2WZD6_ORYSI/57-301

VFSFLRDAIGKDVGLAAALIRLHFHDCFVQGCDASILLTKTPGGPDGEQQAIPNESLRPA

AFKAVNDIRALLDRACGRVVSCSDIVTLAARDSVKLAGGPSYKVPLGRRDGLTSATPSQV

LGALPPPTSHVPELIAALAKLNLDAADLIALSGAHTVGIAHCTSFTGRLYPKQDGTMDKW

FAGQLKLTCPKNDTANTTVNDIRTPNAFDNKYYVDLQNRQGLFTSDQDLFVNATTRPLVA

EFAVD

>Q42854_HORVU/60-300

VRKFVQDAVRKDKGLLRLHFHDCFVQGCDASVLLHGSAAEPGEQQAPPNLTLRPSALKAI

DNIRDQLEHHCHGAVVSCSDILALARDSVVATGGPDYCVPLGRRDSARFATRDAVGSGLP

RPSSNVTTLLDVFRKLGLEATDLVALSGGHTIGLGHCNSFEKRLFPLPDTTMSPSFVARL

KRTCPTMGTDGRPAALDVRTTNVFDNKYFVNLVNQEGLFVSDQDLYTNAITQPIVEHFAR

S

>PER7_ARATH/67-306

VVTKVREWTKSDSSLGPALLRLIFHDCGVTGCDASVLLDYEGTERRSPASKTLRGFELID

DIKSEMEKSCPGKVSCADILTSASRAATVQLGGPYWPNVYGRRDSKHSYARDVEKVPSGR

RDVTALLETFQSYGLNVLDLVVLSGAHTIGKASCGTIQSRLYNYNATSGSDPSIDAKYAD

YLQRRCRWASETVDLDPVTPAVFDNQYYINLQKHMGVLSTDQELVKDPRTAPLVKTFAEQ

>A5AE47_VITVI/21-272

INRKVKEWIDKDYTLAAGLIRLHFHDCAVXGCDASILLDHPGSERWADASKTLRGFQVID

DIKAEVERKCPKTVSCADILTAAARDATILSPAPGDATGLDLVRVPFWMVPYGRKDGRVS

IDKEAQTVPMGXENVTALLEFFQSKGLNVLDLVVLSGAHTIGRTTCGAMQHRLYDFHGTG

EPDPSISPKYLKFLRRKCRWASEYVDLDAITPRTFDVMYYKNLQHNMGLLATDQMLGSDS

RTSDLVATLVSK

>Q5QEB4_URTDI/46-290

VTKAVKKGLKENPRIAPGILRIAFHDCFVRGCDASVLIEGPGTEKTSGANRNIQGYNVID

DAKTELERVCPGVVSCADILTLAARDATVLTGGASWKVPTGRKDGLVSLVAEAGPLPGPR

ENVSEQIRKLDEIGLNTQDLVVLLGSHTLGTTSCALFRFRLYNFTNATESGADPSIDPKF

LPTLRKLCPDGGNGSVRVHLDNRSGEKFDTTFYKNLKRGRGVLQSDQVLWTDLRTQPFVR

RLLDS

>Q4A3Y8_SENSQ/44-286

VQSVVKSAIRTNPTYAPGKLRLFFHDCFVNGCDASVLLDGSTSEQTASTNSHLRGFEVIT

AAKDRVETECPGVVSCADILALAARDSVVETGLPRWEVPTGRRDGLVSRAEDALKLPGSR

DSAEVQIEKFAAKGLNIEELVTLVGGHTIRTSACARFVHRLYNYSNTNAPDPHIDQAFLP

HLQTLCPEHGDITIRVDLDTGSVNNFDTSYYENLRKGRGVLESDTKLWTHHITQNLVQQF

ISV

>A5ADN7_VITVI/166-406

VSSTVVTHFKKDPTIAAGVLKLHFKDCFFQGCDGLVSEIDALTDTEIRGFGVIDDAKTQL

ETLCPGVVSCADILALAARDAVGLSGGPSWPVPTGRRDGRLSFGVSPENLTLPVPTDSIP

VLREKFAAKGLNNHDLVTLIGAHTIGLTDCSSFEYRLYNFTAKGNADPTINQAFLAQLRA

LCPDVGGDVSKKGVPLDKDSQFKFDVSFFKNVRDGNGVLESDQRLFGDSETQRIVKNYAG

N

>Q2R8Z9_ORYSJ/35-281

VASAVRQFADADSTILPALVRLQFHDCFAKGCDGSVLIKGVGNNAEVNNNKHQGLRGLDV

VDSIKQQLESECPGVVSCADIVVLASRDAIAFTGGPSFDVPTGRRDGRTSSLRDADVLPD

VKDSIDVLRSKFAANGLDDKDLVLLSSAHTVGTTACFFLQDRLYNFPLAGGGRGADPSIP

EAFLSELQSRCAPGDFNTRLPLDRGSEAEFDTSILRNIRNGFAVIASDAALYNATATVGV

VDTYSSM

>PER2_ARAHY/48-290

VRSTVRSHVNSDPTLAAKILRMHFHDCFVQGCDGSILISGPATEKTAFANLGLRGYEIID

DAKTQLEAACPGVVSCADILALAARDSVVLSGGLSWQVPTGRRDGRVSQASDVSNLPAPS

DSVDVQKQKFAAKGLNTQDLVTLVGGHTIGTSECQFFSNRLFNFNGTAAADPAIDPSFVS

NLQALCPQNTGAANRVALDTGSQFKFDTSYFSNLRNRRGVLQSDQALWNDPSTKSFVQRY

LGL

>PER17_ARATH/39-285

VRREMKKAMIKEARSVASVMRFQFHDCFVNGCDASLLLDDTPNMLGEKLSLSNIDSLRSF

EVVDDIKEALEKACPATVSCADIVIMAARDAVALTGGPDWEVKLGRKDSLTASQQDSDDI

MPSPRANATFLIDLFERFNLSVKDMVALSGSHSIGQGRCFSIMFRLYNQSGSGKPDPALE

PSYRKKLDKLCPLGGDENVTGDLDATPQVFDNQYFKDLVSGRGFLNSDQTLYTNLVTREY

VKMFSED

>Q682W9_ARATH/46-298

IKKEMECIVKEDPRNAAIIIRLHFHDCFVQGCDGSVLLDETETLQGEKKASPNINSLKGY

KIVDRIKNIIESECPGVVSCADLLTIGARDATILVGGPYWDVPVGREDSKTASYELATTN

LPTPEEGLISIIAKFYSQGLSVEDMVALIGAHTIGKAQCRNFRSRIYGDFQVTSALNPVS

ETYLASLREICPASSGEGDSNVTAIDNVTPNLFDNSIYHTLLRGEGLLNSDQEMYTSLFG

IQTRRIVSKYAED

>B8B2I9_ORYSI/76-330

VRDVVERAVAADPRMAASLLRLHFHDCFVNGCDGSVLLDDKPLFIGEKTAGPNANSLRGF

EVIDAIKAELENACPETVSCADVLAIAARDSVVASGGPSWQVEVGRKDSRTASLQGANTN

LPAPTSGVATLVQKFRNVGLSAKDMVALSGAHTIGKARCTTFSARLAGVGASAGGGATPG

DLSFLESLHQLCAVSAGSALAHLDLVTPATFDNQYYVNLLSGEGLLPSDQALASAGAAAA

GAEDVAGLIAAYAFD

>I1QD71_ORYGL/40-284

IKLVVGAAILKEPRMGASLVRMHFHDCFVNGCDGSVLLDDTDDMIGEKLAKPNNMSLRGF

DVIDAIKVAVNTACLGNVVSCADILAVAARDSIVALGGSSYEVLLGRRDATTASIDDAND

DIPNPFMDLPDLVDNFESHGLSLQDLVVLSGGHTLGYSRCLFFRSRLYNETDTLDPAYAA

ALEEQCPIVGDDEALASLDDTPTTVDTDYYQGLTQGRALLHTDQQLYQGGGGGDSDELVK

YYGEN

>PERX_SOLTU/85-320

VRGVVDSAIDAETRMGASLIRLHFHDCFVDGCDGGILLDDINGTFTGEQNSPPNANSARG

YEVIAQAKQSVIDTCPNISVSCADILAIAARDSVAKLGGQTYNVALGRSDARTANFTGAL

TQLPAPFDNLTVQIQKFNDKNFTLREMVALAGAHTVGFARCSTVCTSGNVNPAAQLQCNC

SATLTDSDLQQLDTTPTMFDKVYYDNLNNNQGIMFSDQVLTGDATTAGFVTDYSND

>Q5JBR4_IPOBA/77-310

VKEIVEAAITNETRMGASLIRLFFHDCFVDGCDGGILLNATNGEQSAPANANSVRGFEVI

ERAKQNAKSKCSDTPVSCADVLAIAARDSVVKLGGQTYTVNLGRRDARSFNLTGANNQLP

APFDDLATQTRKFADKGFNQTEMVALAGAHTVGFARCAVLCSSNNLNQARNSTLQCTCPV

AAGDAGLVGLDPTPSTMDTRYFRDIVDGQGLLFSDQVLLNGTTTTAAVRRYRDG

>Q5I3F2_TRIMO/43-285

VRATMIKALLAERRMGASLLRLHFHDCFVQGCDGSILLDDVGSFVGEKTAFPNVDSVRGY

EVIDEIKKNVELLCPGIVSCADIAALAARDGTFLLGGPSWSVPLGRRDSTTASLTEANSD

LPAPSLSLGLLIKAFDKKQLSPQDLTALSGAHTIGFSQCLNFRDHIYNGTNIDPAFATLR

KRTCPAQAPNGDKNLAPFDVQTQLLFDNAYYRNLVAKRGLLNSDQVLFNGGSQDALVRQY

VAN

>A2YDJ5_ORYSI/53-298

VRSVMERHAAANPRTAPAILRLFFHDCFVNGCDASILLNATDSMESEKDAKPNASVVGYD

VIEDIKSELERSCPATVSCADVLALAARDAVAMLGGPSWGVLLGRKDSLAARMDMANKDL

PRPTDSLAELIRMFKENNLDERDLTALSGAHTVGRTHSCEHYEERIYSLVGQGGDSIDPS

FAAQRRQECEQKHGNATAPFDERTPAKFDNAYYVDLLARRGLLTSDQELYTQGCETGDLV

KTYAMN

>A3A4X6_ORYSJ/53-303

VRSVMAAGGGGRTGAWARPCFRLFFHDCFVNGCDGSVLLDDAPPGFTGGKGRRRDAGSAR

GFEVVDAAKARVEAACRATVSCADVLALAARDAVALLGGTTWPVRLGRKDARTASQAAAN

GNLPGPVSSLTSLLATFAAKGLSARDMTALSGAHTVGRARCATFRGRVNGGDANVNATFA

AQLRRLCPAGTGGDGNLAPLDAETPDVFDNGYFRELTKQRGLLHSDQELFAAGGGGRSSS

QDALVRKYAGN

>I1NYV0_ORYGL/48-289

VRQVMSQAVMNDTRAGAAVLRLFYHDCFVGGCDASVLLDDTPAAPGEKGVGPNAVGSTTV

FDLVDTIKAQVEAVCPATVSCADVLAIAARDSVNLLGGPSWAVPLGRRDALSPSRSAVST

DLPGPEADISALVSAFAAKGLSSRDLAALSGAHTVGRASCVNFRTRVYCDANVSPAFASH

QRQSCPASGGDAALAPLDSLTPDAFDNGYYRNLVAGAGLLHSDQELFNNGPVDSVVQLYS

SN

>PER1_ZINVI/48-285

IRTSIRSSVSSNRRNAALVIRLLFHDCFVQGCDASLLLSGAGSERASPANDGVLGYEVID

AAKAAVERVCPGVVSCADILAVAARDASVAVGGPSWTVRLGRRDSTTSNAAQAATDLPRG

NMVLSQLISNFANKGLNTREMVALSGSHTLGQARCIRFRGRIYNSTLRIEPNFNRSLSQA

CPPTGNDATLRPLDLVTPNSFDNNYYRNLVTSRGLLISDQVLFNADSTDSIVTEYVNN

>PER5_ARATH/42-285

IRSSVRTAIARERRMAASLIRMHFHDCFVHGCDASILLEGTSTIESERDALPNFKSVRGF

EVIDKAKSEVEKVCPGIVSCADIIAVAARDASEYVGGPKWAVKVGRRDSTAAFKALANSG

ELPGFKDTLDQLSGLFSKKGLNTRDLVALSGAHTIGQSQCFLFRDRLYENSSDIDAGFAS

TRKRRCPTVGGDGNLAALDLVTPNSFDNNYYKNLMQKKGLLVTDQVLFGSGASTDGIVSE

YSKN

>A5C5U0_VITVI/1-253

MQWLTSPAWGPHCSAFISMIALYWQGCDASILLDDTANFTGEKTAGPNNNSXRGYDSSNC

GSDWYPLCIRVVTCTENTIKSQMESLCPGVVSCADIVAVAARDSVVALGGPTWTVQLGRR

DSTTASFSTANSDLPAPTSDLDALISLFSNKGFTTQEMVVLSGTHTIGKAQCSKFRDRIY

NETNIDATFATSKQAICPSSGGDENLSDLDXTTTXFDNVYFTNLIEKKGLLHSDQQLYNG

NSTDSMVETYSND

>A4UN76_MEDTR/44-286

VKSTLQTAISKEARMGASILRLFFHDCFVNGCDGSILLDDTSSFTGEKNANPNRNSARGF

DVIDNIKTAVENVCPGVVSCADILAIAAADSVAILGGPTWNVKLGRRDAKTASQSAANTA

IPAPTSNLNTLTSMFSAVGLSSKDLVTLSGAHTIGQARCTNFRARIYNETNINAAXASTR

QSNCPKASGSGDNNLAPLDLQTPSSFDNNYFKNLVQNKGLLHSDQQLFNGGSTNSIVSGY

STS

>PER67_ARATH/37-280

VRRVVKRAVAREPRMGASLLRLFFHDCFVNGCDGSLLLDDTPSFLGEKTSGPSNNSVRGF

EVIDKIKFKVEKMCPGIVSCADILAITARDSVLLLGGPGWSVKLGRRDSTTANFAAANSG

VIPPPITTLSNLINRFKAQGLSTRDMVALSGAHTIGRAQCVTFRNRIYNASNIDTSFAIS

KRRNCPATSGSGDNKKANLDVRSPDRFDHGFYKQLLSKKGLLTSDQVLFNNGPTDSLVIA

YSHN

>Q5W5I3_PICAB/55-304

IKSMVEDAVKKEARIAASLLRLHFHDCFVKGCDASLLLDDNASFTGEKTAIPNKNSLRGF

EVVDKIKSNLEKACPGVVSCADILAVAARDSVAISGGPFWKVLLGRRDSRSASKSGANED

LPAPNSTHQTLETKFKLQGLNVVDLVALSGAHTIGLARCASFKQRLYNQTGNKPDQTLDT

TYLKQLRTVCPQTGTDNNQTRPFDPVSPTKFDVNYYKNVVAGKGLLNSDEILYSTKGSRT

AGFVKYYTTN

>A2WN49_ORYSI/59-308

VVSVLKKAIAKEQRIAASLLRLLFHDCFVQGCDASVLLDDSEEFVSEKKAIPNKNSIRGF

EVIDEIKAALEEACPHTVSCADTIALAARGSTVLSGGPYWELPLGRKDSKAAYMKLANKN

LPPPNATLHRLVKFFERQGLDKVDLVALSGSHTIGMARCVSFKQRLYNQHRDNQPDKTLE

RMFYSTLASTCPRNGGDNNLRPLEFATPSKFDNTYYKLLIEGRGLLNSDEVLWTGRDPQI

AGLVRSYAEN

>A2XTH3_ORYSI/56-307

VGGIVARAHAEDPRMAASLLRMHFHDCFVQGCDASVLLDADGSGRFATEKRSNPNRDSLR

GYEVIDEIKAALEHACPRTVSCADIVAVAARDSTALTGGPWWEVPLGRRDSLTASLSGSN

NLIPAPNDTLPTIVGKFRNQGLDVVDLVALSGGHTIGNSRCVSFRQRLYGQLNSDGKPDF

TLNPAYAAELRERCPSSGGDQNLFALDPASQFRFDNQYYRNILAMNGLLSSDEVLLTKSQ

ETMELVHRYAAS

>PER20_ARATH/46-299

VKHNIEVAVLKDPRMAASLLRLQFHDCFVLGCDASVLLDTHGDMLSEKQATPNLNSLRGF

EVIDYIKYLLEEACPLTVSCSDILALAARDSVFLRGGPWWEVLLGRRDSLKASFAGANQF

IPAPNSSLDSLIINFKQQGLNIQDLIALSGAHTIGKARCVSFKQRIVQPNMEQTFYVDEF

RRHSTFRRVLGSQCKDSSRDNELSPLDIKTPAYFDNHYFINLLEGRGLLISDNVLVSEDH

EGEIFQKVWEYAVN

>Q8S5Y4_ORYSJ/44-296

VRRVVQEARCTDPRAPASLLRLHFHDCFVNGCDGSLLLDDFGAMQSEKNAPPNKGSARGF

DVVDGIKAALENACPGVVSCADILALAAEISVELSGGPSWNVMLGRRDGTAANFEGARDL

PGPTDDLDLLRRKFSEFNLDDTDFVALQGAHTIGRAQCRFFHDRLYNISGTEQPDQTLDM

AYLNELRQSCPASDPESAALRNLDPPTPDAFDNSYYGNLLRNRGLLQSDQGMLSAPGGAA

STTAPIVVWFAGS

>Q18PQ7_PEA/45-294

VREVIRNVSKTDPRMLASLVRLHFHDCFVQGCDASVLLNKTDTVVTEQEAFPNINSLRGL

DVINRIKTAVENACPNTVSCADILALSAQISSILAQGPNWKVPLGRRDGLTANQSLANTN

LPAPFNTLDELKAAFAKQGLTPTDLVALSGAHTFGRSHCSLFVDRLYNFSNTGKPDPSLN

TTYLQELRKTCPKGGSGTNLANFDPTTPDRFDKNYYSNLQVKKGLLQSDQELFSTSGADT

ITIVNKFSAD

>Q43100_POPTR/42-293

IRDVITETLVSDPRIGASLIRLHFHDCFVNGCDGSLLLDNTDTIVSEKEAGGNNNSARGF

EVVDTMKALLESACPATVSCADILTIAAEESVVLAGGPNWTVPLGRRDSTTASRDAANAF

LPAPFFTLDQLRESFTNVSLNNNSDLVALSGAHTFGRAKCSTFDFRLYDFNSTGAPDPSL

DTTLLAALQELCPEGGNGSVITDLDLSTPDAFDSDYYSNLQGNRGLLQTDQELFSTPGAD

DVIALVNAFSAN

>PER38_ARATH/40-291

VTNTIVNALRSDPRIAASILRLHFHDCFVNGCDASILLDNTTSFRTEKDAFGNANSARGF

DVIDKMKAAIEKACPRTVSCADMLAIAAKESIVLAGGPSWMVPNGRRDSLRGFMDLANDN

LPGPSSTLKQLKDRFKNVGLDRSSDLVALSGGHTFGKSQCQFIMDRLYNFGETGLPDPTL

DKSYLATLRKQCPRNGNQSVLVDFDLRTPTLFDNKYYVNLKENKGLIQSDQELFSSPDAA

DTLPLVRAYADG

>Q19MQ5_CUCPE/34-278

VRQEVKRAIETDIRAGAKLIRFHFHDCFVQGCDGSVLLEDAPGIDSELNGLGNLGIQGLE

IVDAIKAAVESECPGVVSCADVLALAAKQSVDVQGGPSWRVLFGRRDSRTANRTGADELP

SPFETLEPLKQKFEALGLDSTDLVAPSGAHTFGRSRCMFFSGRFSNFNGTGQPDPALDPA

YRQELERACTDGETRVNFDPTTPDTFDKNYYTNLQANRGLLTSDQVLFSTPGADTIEIVN

RLGSR

>PER58_ARATH/41-293

ARGLIERASRNDVRLTAKVMRLHFHDCFVNGCDGSVLLDAAPADGVEGEKEAFQNAGSLD

GFEVIDDIKTALENVCPGVVSCADILAIAAEISVALAGGPSLDVLLGRRDGRTAIRADAV

AALPLGPDSLEILTSKFSVHNLDTTDLVALSGAHTFGRVQCGVINNRLHNFSGNSGQSDP

SIEPEFLQTLRRQCPQGGDLTARANLDPTSPDSFDNDYFKNLQNNRGVIESDQILFSSTG

APTVSLVNRFAEN

>Q42964_TOBAC/40-286

VRGVMEQRQRTDARAGAKIIRLHFHDCFGCDGSILLDTDGIQTEKDAIPNVGAGGFDIVD

DIKTALENVCPGVVSCADILALASEIGVALAGGPCWQVLFGRRDSLTANRSGANSDIPSP

FETLAVMTPQFTNKGMDLTDLVALSGAHTFGRARCGTFEQRLFNFSGSGNPDPTVDATFL

QTLQGICPQGGNNGNTFTNLDISTPNDFDNDYFTNLQNNQGLLQTDQELFSTSGSATIAI

VNRYAGS

>A0A0D2S6N2_GOSRA/41-286

VSNVLQQAQGNDIWIFPKIVRLHFHDCFVHGCDASLLLNGTDGEKTATPNLSTEGYEVID

DIKTALEKACPRVVSCADVLALAAQISVSLGGGPKWQVPLGRRDSLTAHREGTGSIPTGH

ESLANIATLFKSVGLDSTDLVALSGVHTFGRARCAAFMDRLYNFNNITGKTDPTLNATYA

NTLKQRCPKGGDTKSLIDLDEQSSLTFDNKYFSNLQNRRGLLQTDQELFSTNGAETVAIV

NRFASS

>Q9FPP5_GOSHI/22-261

FWAQLSATFDYSECPNVTMQSLLRLHFDCFVNGCDGSILFTGEQTAAPNNGSVRGYYVIN

DTLHDGIEAAISEKDASIVGGFDIVPQKTALSWEVKRDSNFTGVGRRDSKTASFNADSGP

TWQVLLGRRDRLTANRSGVDSDIPTPFESLDVMRPQFTNKGMDITDLVALSLSGSHTIDI

GDARCVSFSDRIYNETNIDPTSFDNNSYNNLIEQKGLLHSDQVLFNGGSTDSLVRSYSQS

>A2Q692_MEDTR/72-322

VRREVLNAINEEIRMAASLLRLHFHDCFVNGCDASILLDGDEDIEKFATPNINSARGFEV

IDRIKSSVESSCSGVVSCADILAIVARDSVHLSGGPFWYVQLGRRDGLVSNKTLANNAIP

SPFDSLDTIISKFDNVGLSVKDVVTLSGAHTIGRARCTFFSNRLFNFSGTQEPDNSLEYE

MLTELQNLCPQDGDGNTTTVLDPYSFDQFDNNYFKNLLNGKGLLSSDQILFSSDEETTST

TKQLVQYYSEN

>A2XEA5_ORYSI/51-299

VRSRVAAAMKAEMRMGASLLRLHFHDCFVNGCDASILLDGTNSEKFAAPNNNSVRGYEVI

DAIKADLESACPGVVSCADIVALAAKYGVLLSGGPDYDVLLGRRDGLVANQTGANSNLPS

PFDSISVITARFKDVGLNATDVVVLSGAHTIGRSRCLLFSNRLANFSATNSVDPTLDSSL

ASSLQQVCRGGADQLAALDVNSADAFDNHYYQNLLANKGLLASDQGLVSSSGDPAVAATK

ALVQAYSAN

>Q5U1S5_ORYSJ/80-326

VSSTVRELYLANPNVAAALVRLFFHDCFIHGCDASVLLDRINGDKSEREAAPNQSLRGFG

AVDKIKARLEAACPRTVSCADILVLAARDSLVLAGGPSYPVLTGRSDSARAFYDEVGARI

PSPNATYTVTLDAFARRGFTERETVALLGAHSIGKVHCRFFKDRIDNFAGTGEPDDTIDA

DMVEEMRAVCDGDGAAPMEMGYYRQGREVGFGAHYYAKLLGGRGILRSDQQLTAGSTVRW

VRVYAAG

>A2WN24_ORYSI/42-286

VRQVMERRFYNDNTVAPAIIRMLFHDCFVTGCDASLLIVPTPTRPSPERVAIPNQTLRAL

NIVNAVKSALEAACPGVVSCADALALMARDSVALLGGAAYDVALGRRDALHSNSWEVDLP

APFSSLDDTLRHFAAKGFTADETVLLFGAHTVGAAHCSSFRYRLARPDDSTMDESLRCDM

VGVCGLADQPAAADDAMTFLDPVTPFAVDNAYYAQLMSNRSLLQVDQEAATHAATAGYVA

YYAAN

>A3FPF7_NELNU/48-292

IKEQVKLLYKRHKNTAFSWLRNIFHDCAVQSCDASLLLDSTRRDLSEKETDRSFGLRNFR

YLDTIKEAVERECPGVVSCADILVLSARDGIVALGGPYIPLKTGRRDGRRSRADVIEQFL

PDHNESISVVLDRFAAMGIDTPGVVALLGAHSVGRTHCVKLVHRLYPEVDPALNPDHVEH

MFKKCPDPIPDPKAVQYVRNDRGTPMKLDNNYYRNIMDNKGLLIVDHQLANDKRTKPYVK

KMAKS

>PER21_ARATH/46-291

IRQQVETLYYKHGNTAVSWLRNLFHDCVVKSCDASLLLETARGVESEQKSKRSFGMRNFK

YVKIIKDALEKECPSTVSCADIVALSARDGIVMLKGPKIEMIKTGRRDSRGSYLGDVETL

IPNHNDSLSSVISTFNSIGIDVEATVALLGAHSVGRVHCVNLVHRLYPTIDPTLDPSYAL

YLKKRCPSPTPDPNAVLYSRNDRETPMVVDNMYYKNIMAHKGLLVIDDELATDPRTAPFV

AKMAAD

>Q0U8Y8_PHANO/35-235

VAKELQSTFSTCGRAAHGAIRAPFHDCINNGCDGSLILTDECSRSENGGLSFICETLLDL

TNKYKVSAADMIQFAAAYAISACPLGPNVKALVGRTDSSVAAPLNSMPSSKDSVDSILAA

FSARGFSSDDVVALLGTHSVAIQVVTDPNNVGQPLDSTPAVCDTKYYVETQSGTAPSSLD

SDLRMANDTQTAKTWTDFGNS

>LIG_PHLRA/44-280

VRDDLQNNMFNNECGDEAHEALRLTFHDAIAISPAMEATGQFGGGGADGSIMIFSDIETK

FHPNIGLDEVVESFRPFQQRSGMGVADFIQFSGAVGTSNCPGAPTLNAFIGRKDATQAAP

DGLVPEPFHDVNTILARFNDAGDFDELETVWFLIAHSVAAQNDIDPAVSHAPFDSTPSVM

DGQFFIETQLRGVEFIGSGGIEGVAESPVKGEFRLMSDQQIARDNRTACEWQSFGTD

>Q9UVY8_PLEOS/47-283

ILEDIQTNLFDGAQCGEEVHESLRLTFHDAIAFSPALTNAGQFGGGGADGSMIIFSDTEP

NFHANLGIDEIVEAQKPFIARHNISAADFIQFAGAIGVTNCAGAPRLNFFLGRPDATQIP

PDGLVPEPFDSVDKILSRMGDAGFSTVEVVWLLSSHTIAAADLVDPSIPGTPFDSTPSTF

DSQFFLETMLQGTAFPGTPGNQGEVESPLAGEMRLQSDFLLARDSRSACEWQSMVNN

>Q12170_PHACH/44-285

LAQDLQETLFQGDCGEDAHEVIRLTFHDAIAISQSLGPQAGGGADGSMLHFPTIEPNFSA

NNGIDDSVNNLLPFMQKHDTISAADLVQFAGAVALSNCPGAPRLEFMAGRPNTTIPAVEG

LIPEPQDSVTKILQRFEDAGNFSPFEVVSLLASHTVARADKVDETIDAAPFDSTPFTFDT

QVFLEVLLKGTGFPGSNNNTGEVMSPLPLGSGSDTGEMRLQSDFALARDERTACFWQSFV

NE

>PER_ARTRA/49-285

VLDDLQTNFYQGSKCESPVRKILRIVFHDAIGFSPALTAAGQFGGGGADGSIIAHSNIEL

AFPANGGLTDTIEALRAVGINHGVSFGDLIQFATAVGMSNCPGSPRLEFLTGRSNSSQPS

PPSLIPGPGNTVTAILDRMGDAGFSPDEVVDLLAAHSLASQEGLNSAIFRSPLDSTPQVF

DTQFYIETLLKGTTQPGPSLGFAEELSPFPGEFRMRSDALLARDSRTACRWQSMTSS

>Q5XXE2_PHACH/47-278

VREEIQDRLFNRSCGADVRKSIRIQWHDAIAFSHTGGLKSGGGADGSILAHAIELAYPAN

VGLSDIAERQRALALKHNVSFGDFIQFASAVGLSNCEGAPRLTFLAGRPNASRAADDGLG

PAPWHSADTLLARMAHAGFAPHELVALLAGHSVATQQTVDASVAGTPLDSTPRTFDNAFF

VETQARGTVCPGAALHKGEALSSSPHELRLQSDGALARDPRTAPTWKRFAED

>Q0TWP9_PHANO/64-289

VQSCDFGAKQPGVQKAAQWVRTAFHDAATYDATTKIGGLDASIQYELDRPENLGAALNST

LADISSLVTTRSSAADLLALSLVMSVARCANMRVPLRLGRKDATEAGIKGVPEAHTDLDT

SRKRFATMGMDEADMITLIACGHSIGGVNSVDHPEIVSGPVSPENKASFDTTKGAMDNQV

VLEYLDNSTANPLIVNANDTLNSDKRIFAADGNATMRKLADKAYFK

>A0A0D3FZS7_9ORYZ/102-302

IKSTLATAITAKKELIPSLLTLALNDAMTYDKATKSGGPNGSVRLRPENSGLSAAVDLLV

EAKKEIDSYSKGGPIAFADLIQFAAQSALKLTFVDAAIAKCGGNEEKGRTLYSAYGSNGQ

WGLFDKLFGRQDTQEPDPEGRVPDWSKASVQEMKDKFVAVGLGPRQLAVMSVFLGPDQAA

TEERLIADKDCRPWVEKYQRS

>A4ZYP9_PENAM/19-227

ARRKLRALIAEKSCAPLMLRLAWHSAGTFDVSTKTGGPFGTMKNPAEQAHGANAGLDIAV

RMLEPVKEEFPILSYADLYQLAGVVAVEVTGGPEIPFHPGREDKPQPPPEGRLPDATKGS

DHLRQVFGKQMGLSDQDIVALSGGHTLGRCHKERSGFEGPWTRNPLVFDNSYFKELLTGD

KEGLLQLPSDKTLLSDPVFRPLVEKYAAD

>Q42909_MESCR/13-219

ARRDLVALVQSKNCAPIFLRLAFHDAANFNAADKTGGVNGSLRLQEELGQPPNGGIKVGI

DLIEEVKKKHPTVSYADLYQLAGVVAVGASGGPAIFFVPGRKDTDVADTLNIPNPNGGAD

HLRTVFHQMGLVDKDIVTLSGAHTLGRAHSNISGFDGPFTREPLKFDNSYYVELLKGDTE

GLVKFPTDKVLLQDDVFRPLVEIYAKH

>A5JW31_GALSU/83-294

VRTRLIKLFEQTPCMPIMVRLAWHDAGTYDAQTGTGGVNGSIRFEPELKHGANNGLKIAF

DLLEPIKKEYPDIGYADLFQLASVTAIEFAKGPKIPFRMGRKDATGPDSCPEEGRLPNAE

DHLSQLRRTFHRMGLTDKDITVLSGAHTLGRCHKERSGYEGPWTHQPLEFDNSYFVEILK

PDPDPGLLRLASDLSLLEDSYTRNLVETYAAN

>A4RRF3_OSTLU/4-256

AREAAFAMLDARKCHPIMVRLAWHDAGTFDATAADAWPRCGGANGSIRFDAELAHGANAG

LKKALGYAREIVERFPALSHADAIQLCGACAIESAGGPRIPMKYGRKDSDEPAREGNLPD

AEAPFGDGSKTPGEHLRRVFGRLGFDDREIVALSGAHTIGRAFKERSGTTEYGYGVKNAT

KYTGGCPFSPKGDGDGDFGMPGGASWTSCWLKFDNSYFTEGGSDDKNLLWLSTDRVLHTD

PGFAPHFMRYARD

>Q5ENU9_HETTR/39-308

LEGDLKNLINTLNCNPILVRLAWHDSGTFDQRIQGWPQCGGANGAIRFDPEMNFGANAGL

AKAKGYLDKFVEKYPSLSWADMIQMASAVSIEMAGGPKIPMKYGRVAVTSPDQCVGSASR

EGFEGNAGLPDPIPGGNGKFPCGATGPAAHLRNVFTKKMGFTDQEIVALSGAHTIGRAYK

ERSGTCPFGYMDASASKYSKSSCIVRKDGKAGIGMPGGAAWTKNWLTFDNSYFTKFKEAM

EDDHLLWYPTDECLHQDPAFRPIFMKYAES

>APX7_ORYSJ/95-327

AREDIRELLKTTHCHPILVRLGWHDSGTYDKNIKEWPQRGGANGSLRFDVELKHGANAGL

VNALKLVQPIKDKYPNISYADLFQLASATAIEEAGGPKIPMTYGRIDVTGPEQCPPEGKL

PDAGPSAPADHLRKVFYRMGLDDKEIVVLSGAHTLGRSRPERSGWGKPETKYTKNGPGAP

GGQSWTAEWLKFDNSYFKEIKEKRDQDLLVLPTDAALFEDPTFKVYAEKYAED

>Q2V8E8_9CHLO/10-255

ATAAIKELIAAKACGPILIRLAWHDAGTYDDSIGAAAWPKCGGANGSIRFDPEILHGANA

GLKNALILLEPIKAQFPEVGYADLFQLASATAVEVMGGPTIPMKYGRKDATGPDMCHPEG

NLPAGAAPWPTGGDAAGHLRAVFHRMGLSDQDIVALSGAHCVGRAHASRSGLCHKAETKY

TAAGACPMGTAATGGASWTPEWTKFDNSYFQVVKDPKDEELLALETDTVLFKDPEFLKYA

EKYAED

>Q8LP26_EUGGR/393-620

IRADVAALVAEKGCAPILIRLAWHDAGTYDQQSNTGGPRAVMRFPGGEAEHGSNNGLDIA

RGLLQPIVDKYSWVSTADLWAFASVVATEVSGGPKIPFRPGRRDAVTAKEAVERGRLPDA

TQTTNHLRDVFYRMGMTDEEIVALSGAHTMGRCHAERSGFEGPWTDNPLVFDNSYFKLLL

ERKWTAVTNSVGNLQFQDETGTLMMLTSDLALLMDPSFRKHVERFAAD

>Q5ENU8_ISOGA/15-243

VKSDIRKALVNQKGNSCPLAVRLAWHASGTYSKHDDTGGSYGATMRFPPEKEDGANAGLD

IERDILQEVKRQHPDLSYADIWTLAGAHAIEIAGGPPIEHKLGRTDAQDGSACPAVGRLP

DASQGAEHLREVFYRMGFNDEDIVALSGAHTLGRCHKTRSGFDGPWTHEPLKFDNSYFKN

LLDLEWKPRQWDGPLQYEDPSHTLMMLPTDLALKTDPKFKEYVVAFAKS

>Q4Q3K2_LEIMA/44-272

LRADIEDMISEKLELGPSLIRLAWHEAASYDCFKKDGSPNSASMRFKPECLYAGNKGLDI

PRKALETLKKKYPQISYADLWVLAAYVAIEYMGGPTIPFCWGRVDAKDGSVCGPDGRLPD

GSKTQSHVREVFRRLGFNDQETVALIGAHTCGECHIEFSGYHGPWTHDKNGFDNSFFTQL

LDEDWVLNPKVEQMQLMDRATTKLMMLPSDVCLLLDPSYRKYVELYAKD

>Q2HDY7_CHAGB/87-319

VYNEIANRLEENDEYDDGSYGPVLVRLAWHASGTYDAATGTGGSNGATMRFAPESDHGAN

AGLKAARDFLEPVKKKFPWITYSDLWILAGVCSIQEMLGPKIPFRAGRQDKDVAACTPDG

RLPDAAQAQDHLRNIFYRMGFNDQEIVALAGAHALGRCHSNRSGYEGPWTFSPTVLTNDF

YKLLLDEKWQWKKWNGPKQYEDKKTKSLMMLPADMALVEDKKFKNWVKEYAAD

>A5DM07_PICGU/229-458

IRSVFALESHDDGSLAPIIVRLAWHCCATYDKESGTGGSNGSTMRFLPEMTDEGNYGLDM

ARAALEPVKFKFPRITYSDLWTLAGKVAIEHMGGPTIKWICGRVDCPTDWYVPPNGRLPF

GSKDADHVRKTFERMGFNDREAVALIGCHAIGRCHKRLSGWEGKWTRTPTIFTNAFFRAL

LEEEWVLDTVPETGRHQFYNRDKSLMMLNTDMELLRDEEFRSHVVRYAYD

>CCPR3_YARLI/43-277

VRADLHNILPQKNTTVFKDGTLAPLLIRLAWHSCATYDKYTRTGGSNGATMRYHLEASDE

GNVGLEVARLSLEPIKRKHPWITYADLWILAGVVSIEACKGPSIKWRDGRVDYEDDLLVP

PNGRLPLGGGDASHVRTIFSRMGFNDQETVALIGAHSLGRLHHHRSGFDGPWTSNPAKCD

NEFYKLLLGNVWTLVDSPTGRKQYVNSTGQVMMPSDMSLIEDANFRFWVDQYAVS

>CCPR_CANGA/86-318

VYNLIAERLRDDDEYDNYIGYGPVLVRLAWHSSGTWDKNDNTGGSYGGTYRYKKESQDPS

NAGLENAAKFLEPVKKQFPWISYGDLYTLGGVVGIQELQGPKIPWRSGRTDLPEDMTPDN

GRLPDGDKDANYVRNFYKRLDFNDREVVALLGAHALGKTHLKNSGFEGPWGAANNIFTNE

FYLNLLNEDWKLEKNDAGNLQYNSPKGYMMLPTDYALIQDSNYLKIVKEYAAD

>A5DXH7_LODEL/120-347

AKLAAFPHYDKDDGYYAVLVRMAFHLSGTYSKGDNTGGSYGGTMIFPPEEMDFQNNGLQI

ARSFLDQFLYKYPWISRGDLWTLAGVCAVQECGGPKVEWAPGRVNDNKGVFVPPNGRIPD

GGGDGAYVRKTFARMGLGDRETVALIGAHVLGRCHVHNTGYDGPWGDDVNRFTNDFFQRL

LQKWHIKNWSGRKQYEDDETNQYMMLPTDMSLKTNDYFRKYVEIYAKD

>CCPR_DEBHA/92-321

IAEKVRDQDDADDGAGRYGLLTRLAWHTSGTYKKEDNTGGSYGGTMIYKPESTDGENSGL

NHGRDFLQEFKDKYSWLSHGDLWTLGGVVAVQECGGPKIKWRPGRQDISDKTRVPENGRL

PDASKDADYVKGVFGRMGFNERETVCLIGAHCLGKCHKENTNYDGPWGPSFNMFTNDFFV

RLLQNWHVKKWDGKKQYEDDETNSFMMLPTDMALKEDSSFLKYVKMYADD

>A5JW30_GALSU/90-322

VREAIVKVIEVDDNIAPAMLRLAWHSSGSYDKKTNTGGSDGATMRFSPEKDYAANAGLFR

ARDALEPVKKQFPEITYADLWTLAGAVAVEEMGGPKVAWRPGRRDAVSGQECPPDGRLPD

ADKGTLSGTVQHIRDIFYRMGFNDQEIVALVGAHAVGHTHKQFSGYDGPWTRAPTTFSNE

LFRELLENKWTLRKWNGPDMFEDPTGEIIMLPTDMALTWDKEFRKYVETYAAD

>A8BT29_9DINO/49-325

LYQSISKLMKDSKAFWPADGPQDGDVASYAGLFERLAWHCSGTLRVVNGTATGGCEGARQ

RHWPENEWRDNTNLDKARGLLGQVKHEFGFQISWSDLITFAGTTAIKASGGPAKKFCFGR

IDDVDGKRSIPLGVEGVKECRGNKFCKSNFECPIAFRWPEQDEDDHSQCNLTQPDHRLQA

SHSVGLIYVYPEGPKLKSSAKGFVEKQAHNRSPGLSALEVRDTFKERMGWTDRETVALIG

GGHTLGRTHGNCNLTGTKWASHPYNDVGPFFEAQAGS

>Q9S5Q6_GEOSE/69-395

LKEDLRKLMTESQDWWPADYGHYGPLFIRMAWHSAGTYRIGDGRGGASTGTQRFAPLNSW

PDNANLDKARRLLWPIKKKYGNKISWADLFILAGNVAIESMGGKTIGFGGGRVDVWHPEE

DVYWGSEKEWLASERYSGDRELENPLAAVQMGLIYVNPEGPDGKPDPKAAARDIRETFRR

MGMNDEETVALIAGGHTFGKAHGAGPATHVGPEPEAAPIEAQGLGWISSYGKGKGSDTIT

SGIEGAWTPTPTQWDTSYFDMLFGYDWWLTKSPAGAWQWMAVDPDEKDLAPDAEDPSKKV

PTMMMTTDLALRFDPEYEKIARRFHQN

>KATG1_MYCVP/81-408

FKRDVIDLINTSQDWWPADYGSYAGLFIRMSWHAAGTYRIFDGRGGAGQGSQRFAPLNSW

PDNANLDKARRLLWPIKRKYGNKISWADLIAYAGNAALESAGFQTFGFAFGREDIWEPEE

MLWGQEDTWLGTDKRYGGTNDSDTRELAEPFGATTMGLIYVNPEGPEGKPDPLAAAHDIR

ETFGRMAMNDEETAALIVGGHTLGKTHGAADVNVGPEPEGAPIEQQGLGWKCPFGTGNAG

DTVTSGLEVVWTTTPTKWSNAYLELLYGYEWELTKSPGGAWQFEAKDAEAIIPDPFGGPP

RKPTMLVTDVSMRVDPIYGPITRRWLDH

>KATG_DINSH/67-398

VWDDVDKLLTDSQDWWPADWGHYGGLFIRLSWHAAGSYRLGDGRGGAGTGNLRFEPLNSW

PDNASLEKARRLLWPVKKKYGNALSWADLLVLAGTVAYSNMGLKTFGFAFGRKDIWGPEI

DINWGSDSEILAPTDERVTDVADANSMANPLAASHMGLIYVNPEGVNGTPDPAQTAKYVR

MTFARMAMNDEETAALTVGGHTVGKAHGGTMADKVGADPAGCPVHMQGFGWENPGFDGNA

NTAHTSGLEGAWTSNPTQWDNGYLELLFKYDWEVTKSRAGAFQWEPVNIAEEDMVPDATD

PSIKHNPIMTDADMAMKVDPIYREICERFHKD

>KATG_RHOCA/64-392

LRADLHALMTDSQPWWPADWGHYGGLMIRMAWHAAGSYRAADGRGGGNTGKPARFAPLNS

WPDNVSLDKARRLLWPIKKKYGNAVSWADLILFAGTVAYESMGLKTFGFGFGREDIWAPE

KDVYWGAEKDWLAPSDGRYGDLAKPETMENPLAAVQMGLIYVNPEGVNGQPDPARTALHI

RETFARMGMNDEETVALTAGGHTVGKAHGNGDAKALGPDPEAADVTVRALAGRTRIWAAR

RRRPSPRGSRAPGPRIRRAGTWAISRCSSGHDWELTKSPAGAWQWKPVTIAEEAKPLDAT

DLTTRHDPLMTDADMAMKVDPSTMRSVRS

>KATG2_SHEFN/95-421

VKLDINALLTTSQDWWPSDYSNYGPFFIRMTWHSAGTYRTLDGRGGAGGGQQRFEPLNSW

PDNASLDKARRLLWPIKMKYGEAISWSDLIVLAGNVSLENMGFKTYGFAGGRHDDWEPDM

VYWGPEIEMLASDREDNGGKLQRPLGATHMGLIYVNPEGPKGVPDPLGSAKNIRVAFERM

AMNDEETLALIAGGHTFGKMHGAHKPKDCLGAEPAAAGIEEQGLGWKNKCGKGHSEDTIT

SGLEGAWTQAPTKWTSLYLSNLLTFEWKQTRSPAGAIQWIPTDESLHKAVPDAHVKGKFH

APVMTTADLALKYDPEYRKIAERFLAD

>B9LP48_HALLT/45-373

VKADIEDVMTTSQDWWPADYGTYGPLFIRMAWHSAGTYRTHDGRGGAAGGRQRLPPLNSW

PDNVNLDKARRLLWPVKQKYGRKLSWADLIVLTGNVALESMGFETFGFAGGREDEFKPDD

AVDWGPEDEWESSSAERFDEGGSLDEELGNTVMGLIYVNPEGPNGEPDLEGSAANIRDTF

SHMAMNDKETVALIAGGHTFGKVHGADSGDNLGPDPEDAPIDLQGLGWDNEHGEGKGPDT

ITSGIEGPWNATPTAWDLSYVNNLLSYDWEAERGPGGAWQWTTKNDELDDAAPGVQDPSD

KEDVMMLTTDVALKHDDDFRAVLEEFRDD

>A0A0G2PAI1_FRATU/71-399

LKKDMQDLLTQSQDWWPADFGNYGPFFIRLSWHDAGTYRIYDGRGGANRGQQRFSPLNSW

PDNVNLDKARQLLWPIKQKYGDAVSWSDLIVLAGTVSLESMGMKPIGFAFGREDDWQGDD

TNWGLSPEEIMSSNVRDGKLAPAYAATQMGLIYVNPEGPDGKPDIKGAASEIRQAFRAMG

MTDKETVALIAGGHTFGKTHGAVPEDKVKQAIGPAPDKAPIEQQGLGWHNSYGIGNGDDT

MGSGLEGSWTSTPTFWNHDFLHNLYNLDWKKTLSPAGAHQWTPTNAKPENMVPDAHKLGV

KHKPIMFTTDLALKEDDGFNKYTQEFYNN

>A3IP51_9CYAN/69-418

LKKDIKILLTDSKDWWPADYGNYGPQMIRMAWHSAGTYRIADGRGGASRGMQRFAPINSW

WDNGNTDKSRRLLWPIKQKYGAALSWADLMILVGNCALEIMGLKTFGYGGGRIDAWEADR

ATYWGPEFWNGQAFGENGKQHEGHPDEMVTRTIRWVGEPKDEYYDLENPVAASHQALIYV

DPEGPGGNGDPIASARDIRETFARMAMNDEETVALIAGGHAFGKSHGMVAPDKIGPAPEG

APIQAMGLGWQNPEGTGFAEYTMTNGIEGSWTPNPTQWDNSYLENLFKYEWEKTKSPAGA

VQWQPRDPSAPKTPDAHKSGVEHSLMMMTSDIALKEDSAYRKVCQRFLDD

>KATG_PSELT/54-378

VIEDLKKLMKTSQDWWPADFGHYGPLFIRLSWHSAGSYRIHDGRGGAKNGSIRFPARINW

PDNINLDKAIRLLWPIKKKYGKKLSWADLIILAGTVALQDMGVKILGFSLGREDVFEADE

SPDWGAEQEMLSGKERFKEGELEKPFAATEMGLIYVNPEGPMGNPDPSGSAKEIRLAFTR

MGMNDEETVALIAGGHSFGKCHGAGPSKDLGPDPSSSPIEQMGLGWKYTYKTGKASDTYT

SGFEVIWSSKPTKFGIQYLKFLLEFEWELEKSPDGKNQWVAKNAPEMIPDPFDPNKKHKP

RMLTADLALKFDPIYSKIAKKFLEN

>KATG_ARCB4/400-708

ADAFARAWFKLTHRDLGPKSKYIGPEIPKEDLIWQDPIPPINYEIIDEKDIEILKEKLLS

SSLGVSKLVSLAWASASTYRDSDKRGGANGARIALEPQRSWESNSYLNLDESLKILETIK

GEFNSSNSNKKVSLADLIVLGGCAAVEKAAKDAGFNIKVPFTAGRADATQEQTHVESFSH

LEPIADGFRNYSKAKYTLSTEELLIDKAQLLSLTIPEMIVLVGGMRVLGANYANSDLGVF

TSNVGVLSNDFFVNLLDMKTAWYPTTQEEDSFVGKDRQSGSMKYSASRVDLLFGSNSQLR

AVSEVYAQE

>Q26C31_FLABB/440-746

DAAFAKAWFKLTHRDMGPSSTYLGPEVPQEQFIWQDPIPARDYKLVSTSDINTLKGKIKA

SGLTTNELVTTAWNAASTYRHGDRRGGANGGRIQLEPQVNWDSNNPTQLKKVLNVYRDIK

NDFDTSSRKISMADLIVLGGNVAIENAAKKAGYSIDVPFTPGRTDATQDQTDIDGTNLLK

PMADGFTNFQQKEYTLTAEQLLVDKAQQLTLSAPEMTVLVGGMRALGANYDGSKTGIMTE

QTGTLSNDFFKNLLSMDYSWKPVENNKNLFEIVERNTNNKKWDATRVDLIFGSNSELRAL

SEVYASE

>KATG_ARCFU/383-700

EKAFAIAWYKLTHRDMGPKDCYIGKYVPEETFVWQDPLPRRDYELVDEKDVEELKRRILA

SGLSLSQLVYFAWASASTYRNSDRRGGANGARIRLKPMSVWEVNHPEELKKVIAAYEKIQ

QEFNEGAKGSEKRISIADLIVLGGIAAVEEAARRAGFSVKVPFIPGRVDAQQEHVDEEFY

RVIEPFADGFRNYFRYPERINERDVYTTPEYFLVDKANLLTLTVPEMVVLIGGMRALGAN

YSHSDYGVLTERPGVLSNDFFVNLLDMSVEWRAADDYRYTFEGYDRKSGELRWRATRVDL

ILGHHDELRAVAEVYGCD

>A6DDL6_9PROT/71-368

EEAFAKAWFKLTHRDLGPKSRYLGNDIGEDDFIWQDLIPKSYTSISEKEIETLKKEILEK

LSIQEILYTVFSSVSTYRDTDKKGGLNGARIRLKPQIDWEINQDKRVAKTIKTLEKIKPD

NITFADLLALSGIIAIEKAAKNAEVEINLDFYPGRGDATQEETDIESFNYLKPIANGFLN

YLQNGCEELISERALIDLANKLTLTVPEMTVLIGGLRTLGINYNDTPYGRFSEDENRLDN

EFFTKLLDMNIEWKPKNKQVFEGFDRKTNKKVYEATRVDLIFGANSHLRAQSEFYAQD

>Q0FXM4_9RHIZ/425-736

TDAFSRAWFKLVHRDMGPKVRYLGPEVPEEDLDWQDPIPDLDHEVVNESDIATLKSKMMN

AGLSVSDLVFVAWSSASTYRDSDKRGGANGGRIALEPQSGWEVNDPGRTEKVLKALRLIA

DEFNDAQSGNKRISLADAVVLGGCAAVEKAAKDAGVDVTVPFTPGRMDTTQELTNVEFFE

WLQPVSDGFRNYHREDIVYNVPPEQLFLDRAMLLSLTAPEWTALVGGLRVLDTNWDGSKH

GVFTDRPGTLTNDFFVNLTSPAYTWKKADEDGMSFTLNDFKTNEVKFTATRCDLIFGANS

QLRQIAEVYGAN

>Q50484_MYCTX/405-714

ADEFAKAWYKXIHRDMGPXXRYLGXLVPKXTLLWQDPVPAVSHDLVGEAEIASLKSQILA

SGLTVSQLVSTAWAAASSFRGSDKRGGANGGRIRLQPQVGWEVNDPDGDLRKVIRTLEEI

QESFNSAAPGNIKVSFADLVVLGGCAAIEKAAKAAGHNITVPFTPGRTDASQEQTDVESF

AVLEPKADGFRNYLGKGNPLPAEYMLLDKANLLTLSAPEMTVLVGGLRVLGANYKRLPLG

VFTEASESLTNDFFVNLLDMGITWEPSPADDGTYQGKDGSGKVKWTGSRVDLVFGXNSEL

RALVEVYGAD

>KATG_HALSA/385-694

QQSFSKAWYKLIHRDMGPSERFLGPEVPEETMIWQDPLPDADYDLVDDEAVAALKSELLE

SELSIPQLVKTAWASASTYRDSDKRGGANGARIRLEPQRSWEVNEPEQLEAALSTYEDIQ

AEFNDARSDDMRVSLADLIVLGGNAAIEQAAADAGYDVDVPFEPGRTDATPEQTDVESFE

ALKPKADGFRNYLGDDAEREPEELLVDKAELLNLTADDMTVLVGGLRALGVTHGDSELGI

FTDQPGTLTNDFFTTLLDMDYEWEAASEDREVFELRDRETGDVEWTGSRVDLLFGSNTRL

RAIAEVYGSD

>A4C5D4_9GAMM/404-711

ELAFAKAWFKLNHRDMGPRARYLGSEVPKEILLWQDYIPEVNHPLVNEKDITSLKNAILS

SGLTVPELVRVAWGSAASYRDTDMRGGANGARIRLAPQNEWAVNNPAEVSKVLAKLEAIQ

TQFNKKSSKRKISLADIIVLAGASAIEQAAKEAGHTVKVPFTPGRMDASQEMTDVESFKV

LEPKADAFRNFYSEQSYFSPAEMLIERADLLNLTVPEMTVLLGGMRALNANTNGAQHGVL

TAKPGTLNNDFFVNLLDMSTKWTKSGTEGVYEGHDRKTNQLKWTATPVDLIFGSNSELRS

MAEVYAAQ

>A0A0B6KKK7_FRATL/404-714

KEEFAKAWFKLTHRDMGPKSRYIGPWIPEQNFIWQDPVPAADYKQVSTQDIAQLKQDIIN

SGLTNQQLIKTAWDSASTYRKTDYRGGSNGARIALAPEKDWQINEPAKLEVVLTKLKEIQ

TNFNNSKTDGTKVSLADLIVLGGNVGVEQAAKQAGYNIQIPFVPGRTDATQAQTDIESFN

YLKTKSDGFINYTDGSVNADKLPQALVEKASMLNLNIPEMTVLVGGMRALNVNYDNSQEG

VLTTTPGQLNNSFFVNLLDMSTQWKKSDKKDGEYIGIDRKTGKQKWTASPVDLIFGSNSE

LKAVAQVYAEN

>KATG_YERPE/399-709

KMAFARAWFKLTHRDMGPAARYLGDEVPKETFIWQDPLPAANYKMIDSADISELKDKILK

TGLSDTKLIKTAWASASTFRGTDFRGGDNGARIRLAPQKDWPVNDPAELHSVLAALMEVQ

NNFNKDRSDGKKVSLSDLIVLGGNAAIEDAAKKAGYSISIPFTPGRTDASQEETDVSSFA

VLEPTADGFRNYYDAKRNTLSPIASLIDRANKLELTVPEMTVLIGGLRVLDVNSGGSKAG

VLTNTPGQLNNNFFVNLLDMSTKWTKSPKAEGYFDGYDRKTGKLKWTASSVDLVFGSNPE

LRAVAEVYASD

>KATG2_MYCS2/414-722

SEAFAKAWYKLLHRDMGPISRYLGPWVAEPQLWQDPVPAVDHPLVDDQDIAALKSTVLDS

GLSTGQLIKTAWASAASYRNTDKRGGANGARVRLEPQKNWDVNEPAELATVLPVLERIQQ

DFNASASGGKKVSLADLIVLAGSAAIEKAAKDGGYNVTVPFAPGRTDASQENTDVESFAV

LEPRADGFRNYVRPGEKVQLEKMLLERAYFLGVTAPQLTALVGGLRALDVNHGGTKHGVF

TDRPGALTNDFFVNLLDMGTEWKTSETTENVYEGVDRKTGQLKWTATANDLVFGSHSVLR

AVAEVYAQS

>Q0FAJ6_9RHOB/397-684

ADAFSKAWYKLLHRDMGPKIRYQGPEVPEEEMIWQDPVPVGSTNYDVSAIRREIKSCGLS

TLELVETAWASASTFRGTDMRGGANGARISLSPQKDWAVNKPEQLARVLNILKPIAESHG

ASLADVIVLGGGVGIEMVSDVQVAFTPGRGDALAEQTDEESFSVLEPMADGFRNYQKTQF

TVSPEEMLLDRAHLLGLTASEMTVLVGGLRSIGLSDCGHGVWSDDSNLNTGWFNTLLDMN

VEWSPTGPNSYEAKDKKSGEVVRTASRVDLVFGSNSELRALAELYAQE

>KATG_JANSC/403-708

ADAFARAWYKLTHRDMGPIQRYLGNDVPSEELLWQDPVPMPQGPQVNDDEQAELKAAVAA

TGLTAAELVRVAWGSAASYRDSDKRGGANGARIRLQPARGWTVNNPEELDKVLPVLDSIA

DAFNGRGGTQITMADMIVLAGGVGVEMAAREAGHNIHVPFTPGRGDATQEQTDVDSYDVL

EPTSDGFRNYHATFSLREPAEMLVDKAALLGLTAPEMTVLVGGLRAIGATHGGARHGVLT

ETPGALNNAFFKNVLSMDTVWNQTDSAILEGKDRASGQVKWTATIVDLVFGSNSQLRAVA

EVYASA

>A3JTQ9_9RHOB/405-706

AEAFARAWFKLTHRDMGPKILYKGAEVPSEELIWQDPIPANANPELSAADISALKAGILA

TDLSVGDLVSAAWASAASFRGSDKRGGANGARVLLAPQNTWAANDPDRVAKVVDVLKGVQ

AASSKTISLADLIVLGGTAAVEQAAKDAGHTVEVAFSQGRGDATQAQTDAESFDPMEPMS

DGFRNYLAKEFAVPAEHLLVDRAQLLTLSAPEMTALVGGMRVLGTNTDGSAHGVLTQNVG

ALSNDFFVNLLDMATEWKDAGDGTFEGRDRASGDVKWTGTRADLVFGSNSQLRALAEVYA

QD

>KATG_ERYLH/407-709

DDAFARAWFKLCHRDMGPKVRYQGPEVPSEDLIWQDPVPSGTAPSDSDVSSFKSAVLDSG

LTVSELVKAAWASASTYRNSDHRGGANGARVRLAPQKDWAANDPEELGKVLSKLDELRGN

LSMADAIVLAGSAAIEKAARDAGHSVSVDVTTGRGDATDEHTDAESFEPLEPFADGFRNY

LKTKASVKTEEMLIDKAHLLGLSIPEMTALVGGMRALGAVSRHADHGDRIGVLTDRPGQL

TNDFFVNLLDMGTKWEVVDESGDEEFVGKDRKSGDEKWRATRTDLVFGSNSQLRAQAEVF

AES

>Q05R68_9SYNE/408-717

ADCFARAWFKLTHRDLGPKALYLGADVPNETLIWQDPLPIVDHPLVNSDEQNELKQDLLS

LGLSVGELVSTAWASASTFRNSDRRGGANGARIRLQPQNNWEVNDPAQLDRVLGALQDLQ

SRFNRSNPSGAQISLADLIVLGGNAAVEQASRDAGHIIEVPFQAGRVDAGEEHTDAASFN

VLKPMADGFRNWQRQGLPIRAEELLLDRAQQLTLSAPEMTVLLAGLRVLGANSGGNLEGV

FTHRIGVLSTDFVVNLLDMDTTWAPTDQTNNHYEGRDRDTGALRWRASRADLVFGSNSQL

RAIAEVYAQR

>KATG3_MYCS2/406-718

ADTFARAWFKLTHIDMGPIQRYLGPLVPTERMIWQDPVPHVDHELADADDVAALKREILG

SGLSVSQLVTTAWASASTFRNSDKRGGANGARIRLEPQRSWAVNEPEKLAIVLDRLERIR

RRFNDSHRGGKQISAADLIMLGGCAAVEHAAAEAGHPIEVPCRLGRTDAPQEWTDIEWFS

ALEPTADAFRNYVGEGNRPPPEHLLVDRASQLTLTAPQMTVLLGGLRVLGANHGGSPLGV

FTASPGALSNDFFVNLLDVNIEWTPRADTADWTAAYEGRDRRTGEVTWIASRVDLSFASD

PVLRAISEVYASA

>KATG_BLUGH/400-703

ADAFARAWFKLLHRDMGPRSRWLGPEIPKEELIWEDPIPEIDHPIISQEDINNLKKEILS

SGVGHNKLIQTAWASASTFRGGDKRGGANGARIRLAPQKDWKVNNPPQLTCVLETLGKIQ

SSFNSSQTGGKIVSLADLIILAGCAALEKAAGVPVPFSPGRADASQEQTDIKSFSNLEPV

ADGFRNFGRSTPRARAEHMLVDRAQLLTLTPPELTALVGGLRVLDTNFDGSSCGVFTKRP

GQLTNDFFVNLLDPAISWKGIDEDEFFEGIDRKTDEKKWIGSRADLVFGSQAELRAIAEV

YGSA

>KATG1_LEGPH/400-695

AEVFARAWFKLTHRDMGPKTRYIGPDVPKEDLIWQDPVPAGNRAYDIAAAKAKIAASNLT

IGEMVSTAWDSARTFRGSDKRGGANGARIRLKPQKDWEGNEPQRLTKVLQILEDIATDTG

ASVADVIILAGNVGIEKAAKAAGFDIIVPFAPGRGDATDDMTDAESFDVLEPLHDGYRNW

LKKTYDVRPEELMLDRTQLMGLTAHEMTVLVGGLRVLGTNHNNTQYGVFTDRVGALTNDF

FVNLTDMANVWIPSKDNLYEIRDRKAGNIKWTATRVDLVFGSNSILRSYAEVYAQD

>APX6_ARATH/101-304

MQNEIRKVVTKGKAAGVLRLVFHDAGTFELDDHSGGINGSIAYELERPENIGLKKSLKVL

AKAKVKVDEIQPVSWADMISVAGSEAVSICGGPTIPVVLGRLDSAQPDPEGKLPPETLSA

SGLKECFKRKGFSTQELVALSGAHTIGSKGFGDPTVFDNAYYKILLEKPWTSTSKMTSMV

GLPSDHALVQDDECLRWVKRYAED

>A4S0W6_OSTLU/10-228

VRRAIRAALEANVQKTKCPAVLRLVFHDAGTYLASAKDGGMNASVRYELNRPESFGLKRG

LNVVKSAYDALDDTAAAGKVSFADMIACAGAYAVEFTGGPAFLERVPLGRIDVETADPEN

RMPEQTLGGKEMREHFARSGITTRDMVALAGAHTIGGKGFGDAYTFDNAYYATLAADPWH

KANMTKDEAEMAEHIGLPSDKYMREDAESMEWIRKYAND

>APX1_HYDVD/36-287

LIESVKRGDDLPMIAGTVRLAFHDCIGKGKCDGCIDHSKPGNAGLKRVTDRLDALYDASY

KGKISRADFYALASVTALTRSTANLSDKYNGLRKFKVGRKDCSTSPVESIDSSDIPRGSD

GTSKTLQFFKSEFGMKTQEAVALLGAHTLGRCSLQNSGFVGSWVDQRFSTAPPGEENLSP

TSILDNAYYRMIIDIVPWTQVNINGTRIQWQEPSNSIPNDKLPESKRSPLLLNSDMAISW

IIKPSDALGTVS

>Q8H958_MARPO/49-293

VNQWLVANVFTDPTGPAALVRLVFHDCQVNGCDGSVLLDTQPGAVSELESDANFGIRDLR

FIDSIKAAVELACPGVVSCTDILALAARDCVRLTGGPSIRIPLGRKDGRSASNLAADRQL

PPSDISVPAFLSEFAQMGMTADEAVAIIGAHTIGVGHCVNVVNRLFPQQDPALSPLMAGQ

LLTQCPTPNAAFLNNNTILSNDFTNFVFDNQYYRDVMNGNGLFKIDSLIGQNPTTAGIVA

RFAAN

>A0A0E0PJ99_ORYRU/26-271

VDMALAPVFAVDQTSPAALLRLFFHDCQVQGCDGSILLNSDERRNITSELGSDKNFGIRD

VSTIGLVKAAVERACPGQVSCADIVVLAARSAVAHAGGPRIRGVPLGRRDATAASAERAD

AMLPDSFLGIDGALAMFQSKGMTVEETVAILGGHTLGGGHCATVDTARRGRGRSDAAFEA

ALRLACPAAAPRAVAAAVPVLSDATPSWFDNLYYWNAASGRGIFAVDAEEAADARTAGHV

RRFAAD

>PER29_ARATH/54-303

VRSSLSSMFILDPTSPAALLRLMFHDCQVQGCDASILLEPIRDQQFTELDSAKNFGIRKR

DLVGSIKTSLELECPKQVSCSDVIILAARDAVALTGGPLISVPLGRKDSLSTPSKHVADS

ELPPSTADVDTTLSLFANKGMTIEESVAIMGAHTIGVTHCNNVLSRFDNANATSENMDPR

FQTFLRVACPEFSPTSQAAEATFVPNDQTSVIFDTAYYDDAIAGRGNLRIDSEIGADPRT

RPFVEAFAAD
